# Development of an anti-tauopathy mucosal vaccine specifically targeting pathologic conformers

**DOI:** 10.1038/s41541-024-00904-1

**Published:** 2024-06-15

**Authors:** Wenzhi Tan, Jayalakshmi Thiruppathi, Seol Hee Hong, Sao Puth, Sophea Pheng, Bo-Ram Mun, Won-Seok Choi, Kyung-Hwa Lee, Hyun-Sun Park, Duc Tien Nguyen, Min-Cheol Lee, Kwangjoon Jeong, Jin Hai Zheng, Young Kim, Shee Eun Lee, Joon Haeng Rhee

**Affiliations:** 1 Clinical Vaccine R&D Center, Chonnam National University, Hwasun-gun, Jeonnam 58128 Republic of Korea; 2 Department of Microbiology, Chonnam National University Medical School, Hwasun-gun, Jeonnam 58128 Republic of Korea; 3 Combinatorial Tumor Immunotherapy MRC, Chonnam National University Medical School, Hwasun-gun, Jeonnam 58128 Republic of Korea; 4 National Immunotherapy Innovation Center, Hwasun-gun, Jeonnam 58128 Republic of Korea; 5 Department of Pharmacology and Dental Therapeutics, School of Dentistry, Chonnam National University, Gwangju, 61186 Republic of Korea; 6 School of Biological Sciences and Technology, Chonnam National University, Gwangju, 61186 Republic of Korea; 7 Department of Pathology, Chonnam National University Medical School, Hwasun-gun, Jeonnam 58128 Republic of Korea; 8 Department of Pharmacology, Chonnam National University Medical School, Hwasun-gun, Jeonnam 58128 Republic of Korea; 9 Seegene Inc, Seoul, 05548 Republic of Korea; 10 Department of Oral Pathology, School of Dentistry, Chonnam National University, Gwangju, 61186 Republic of Korea; 11 Present Address: School of Biomedical Sciences, Hunan University, Changsha, Hunan 410082 China

**Keywords:** Adjuvants, Alzheimer's disease

## Abstract

Alzheimer’s disease (AD) and related tauopathies are associated with pathological tau protein aggregation, which plays an important role in neurofibrillary degeneration and dementia. Targeted immunotherapy to eliminate pathological tau aggregates is known to improve cognitive deficits in AD animal models. The tau repeat domain (TauRD) plays a pivotal role in tau-microtubule interactions and is critically involved in the aggregation of hyperphosphorylated tau proteins. Because TauRD forms the structural core of tau aggregates, the development of immunotherapies that selectively target TauRD-induced pathological aggregates holds great promise for the modulation of tauopathies. In this study, we generated recombinant TauRD polypeptide that form neurofibrillary tangle-like structures and evaluated TauRD-specific immune responses following intranasal immunization in combination with the mucosal adjuvant FlaB. In BALB/C mice, repeated immunizations at one-week intervals induced robust TauRD-specific antibody responses in a TLR5-dependent manner. Notably, the resulting antiserum recognized only the aggregated form of TauRD, while ignoring monomeric TauRD. The antiserum effectively inhibited TauRD filament formation and promoted the phagocytic degradation of TauRD aggregate fragments by microglia. The antiserum also specifically recognized pathological tau conformers in the human AD brain. Based on these results, we engineered a built-in flagellin-adjuvanted TauRD (FlaB-TauRD) vaccine and tested its efficacy in a P301S transgenic mouse model. Mucosal immunization with FlaB-TauRD improved quality of life, as indicated by the amelioration of memory deficits, and alleviated tauopathy progression. Notably, the survival of the vaccinated mice was dramatically extended. In conclusion, we developed a mucosal vaccine that exclusively targets pathological tau conformers and prevents disease progression.

## Introduction

Tauopathies are a class of neurodegenerative diseases associated with the pathological aggregation of microtubule (MT)-associated tau protein in the human brain, which leads to neurofibrillary degeneration and dementia^1–3^. Tauopathies comprise more than 20 neurodegenerative diseases classified as primary and secondary tauopathies. Primary tauopathies are primarily caused by accumulation of abnormal tau proteins in neuronal and glial cells, while secondary tauopathies accompanies extracellular deposition of other aggregated proteins such as amyloid-β (Aβ), α-synuclein, or TAR-DNA binding protein 43^4^. Alzheimer’s disease (AD), a secondary tauopathy, is the most common cause of dementia and affects more than 35 million people^5–7^. AD is closely related to insoluble hyperphosphorylated tau aggregates, which form neurofibrillary tangles (NFTs)^8^. In addition to the intraneuronal accumulation of tau NFTs, another classical hallmark of AD is interneuronal Aβ deposition^9,10^. The Aβ amyloid cascade hypothesis states that Aβ deposition in the brain initiates the disease process accompanying NFT formation and represents the first neurotoxic insult^11,12^. However, clinical trials aimed at reducing Aβ levels have failed or have shown limited efficacy in improving cognition, highlighting the need for alternative targets^13^. The accumulation of NFTs and the transcytosis-mediated spread of NFTs in the brain are more directly correlated with neuronal loss and progressive cognitive decline in AD^14,15^. Hence, preventive and therapeutic approaches targeting tau pathology progression could serve as promising alternatives^16^. More than 30 drugs that interfere with the aggregation, processing, and accumulation of tau have undergone clinical trials during the last two decades without showing notable clinical efficacy^4^.

Within the field of AD research, interest in tau-targeted immunotherapy is growing immensely. The first vaccination approach in wild-type mice using full-length tau protein emulsified in complete Freund’s adjuvant resulted in tauopathy-like abnormalities and encephalitis^17^. On the other hand, active and passive immunotherapies targeting phosphoepitopes of tau or tau aggregates have been reported to effectively reduce tau pathologies and improve cognitive performance in transgenic animal models^18–22^. However, AD brain tissues contain paired helical filaments (PHFs) that are phosphorylated at numerous serine, threonine, and tyrosine residues and undergo multiple posttranslational modifications^23^. Targeting a limited number of phosphorylated domains may result in insufficient coverage. Moreover, those phospho-tau epitopes are also found in physiological tau proteins^24^. In this regard, sarkosyl-insoluble PHF-tau, a pathological conformer of tau, was experimentally tested in an aged tau-transgenic mouse model^25^. Even insoluble PHF-tau prepared from pathological tauopathy tissue still carried a risk of inducing autoimmune reactogenicity after vaccination^25^. In this context, we hypothesized that the pathological conformation formed by the repeat domain of tau (TauRD) only could serve as an effective vaccine target.

Despite the impermeability of the blood-brain barrier (BBB), multiple investigations have demonstrated the translocation of plasma immunoglobulins to brain tissue and their predominant internalization into neurons, facilitated by low-affinity Fc receptors^26–30^. In various neurological disorders, including AD, immunoglobulins gain access to the central nervous system (CNS) by crossing compromised BBB^31,32^. Moreover, IgG can traverse the BBB through adsorption-mediated transcytosis^33^. If effective antibody responses against pathologic tau conformers are induced, the prevention and resolution of PHF-mediated tauopathies should be possible. Undoubtedly, selection of safe and effective epitope should be the key to the successful generation of therapeutic antibodies. Recent studies have suggested that soluble tau oligomers, rather than insoluble aggregates, are likely the toxic species most responsible for neuron dysfunction and death^34,35^. Moreover, increased levels of tau oligomers have been detected in the early stages of AD, before NFT formation and the manifestation of clinical symptoms^36^. Therefore, the specific targeting of prefibrillary tau oligomers should be the key to successful AD immunotherapy. Physiological tau is a highly soluble, natively unfolded protein that stabilizes microtubules along the axonal transport track^37,38^. The hyperphosphorylation of tau is the major trigger that induces assembly into PHFs, which precedes aggregation into NFTs^39,40^. TauRD, which includes three to four pseudo-repeats, is responsible for tau–tau–microtubule interaction, and the hexapeptide motifs ^275^VQIINK^280^ and ^306^VQIVYK^311^ in the four-repeat domain have been shown to initiate PHF assembly with a high tendency to form β-sheets^41^. TauRD constitutes the majority of the microtubule-binding region in both 3R and 4R isoforms of physiological tau^4^. In vitro studies have also indicated that TauRD can assemble much faster than full-length tau into PHFs, and this rapid assembly is independent of phosphorylation^42^. Furthermore, a truncated tau polypeptide containing the repeat domains has been found to build the core of PHFs in Alzheimer’s brains^43–45^. The most advanced anti-tau vaccine, AADvac1, which reached phase 2 clinical trial, targets a selected part of TauRD (^294^KDNIKHVPGGG^305^) in an attempt to differentially recognize the pathological conformation of tau aggregates^46,47^. This epitope was selected from a study identifying binding epitopes of the pathological tau-specific monoclonal antibody DC8E8, which exhibited no off-target binding^48^. Considering the difficulty of extracting defined soluble oligomers from the brain and other tissues, TauRD could serve as an excellent protective epitope for AD immunotherapies.

Mucosal vaccination can trigger both humoral and cell-mediated protective immune responses in both mucosal and systemic compartments and has the advantages of reduced costs, improved accessibility, needle-free delivery, and self-administration^49,50^. Positive results have also been reported after repeated mucosal (intranasal) administration of the Aβ peptide to transgenic mice, which induced antibodies against Aβ and achieved partial clearance of Aβ plaques^51,52^. Mucosal vaccines require appropriate adjuvants, as mucosally administered antigens are generally less immunogenic than those administered through systemic routes^53^. Flagellin, the structural component of flagellar filaments, is a cognate TLR5 ligand with strong mucosal adjuvant activity^54–56^. In our previous studies, we repeatedly confirmed that the *V. vulnificus* flagellin FlaB is a potent mucosal adjuvant^54,55,57–62^. Intranasally administered FlaB was mostly trapped by mucosal dendritic cells, which subsequently mobilized to the parafollicular area of cervical lymph nodes and stayed there for a considerable duration^54,57^. Brain antigens, similar to nasally administered vaccine antigens, are reported to drain into cervical lymph nodes through the CNS lymphatic system^63,64^, which suggests that nasal immunization would have advantage for inducing immune responses against CNS-derived antigens. We hypothesized that FlaB can serve as an effective mucosal adjuvant for TauRD-based AD vaccines. In this study, we found that intranasal immunization with TauRD formulated with FlaB can induce the production of IgG antibodies that specifically recognize TauRD oligomer β-sheet aggregates while saving TauRD monomers, which would result in the prevention and resolution of pathological PHF-tau in the tauopathic brain.

## Results

### Development of pathologic tau conformer antigen

**Fig. 1:**
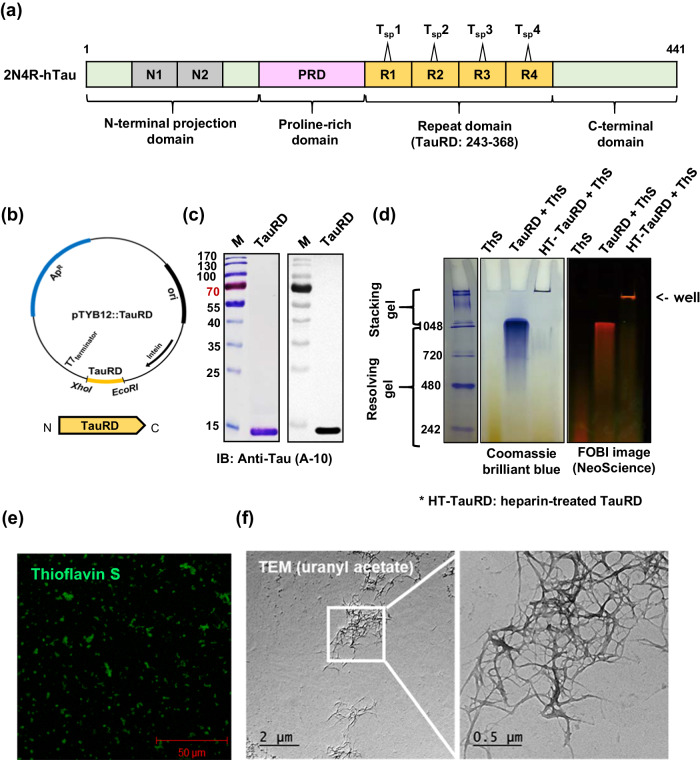
Development of the TauRD antigen. **a** Schematic representation of human 2N4R-tau with annotation of functional domains (TauRD) and four Tau short peptides (Tsp). The diagram illustrates the structural organization of human 2N4R-tau, with the amino acid residues indicated by numbers 243-368 representing the recombinant TauRD polypeptide. **b** Construction of an expression vector for the TauRD antigen. The DNA fragments representing the tau repeat domain (TauRD), the intein tag gene, the T7 promoter region, the ampicillin resistance gene (Ap), and the cloning restriction enzyme sites are depicted in this figure. **c** Analysis of purified recombinant TauRD by SDS-PAGE and Western blotting. Recombinant TauRD was resolved using SDS-PAGE, and the TauRD protein bands were detected and visualized using a tau (A-10) antibody obtained from Santa Cruz Biotechnology (cat. sc-390476). **d** Native-PAGE analysis of purified recombinant TauRD and heparin-treated TauRD aggregates. Twenty micrograms of TauRD or heparin-treated TauRD fibrils were incubated with 3.1 mM thioflavin S (ThS) for 15 min at room temperature, maintaining a TauRD:thioflavin S ratio of 5:1. The samples were subsequently mixed with native loading buffer (comprising 60 mM Tris-HCl, 25% glycerol, and 0.1% bromophenol blue) and loaded into a 10% native-PAGE gel. Gel imaging was performed using a fluorescence-labeled organism bioimaging instrument (FOBI; NeoScience). **e** Confocal microscopic observation of heparin-treated (5 days) TauRD aggregates with thioflavin S staining. This figure shows the results of confocal microscopy observation of aggregated TauRD. Thioflavin S staining was employed to confirm the presence of a β-sheet structure within the TauRD aggregates. **f** Transmission electron micrographs of TauRD proteins aggregated via heparinization. The fibrillization of TauRD was initiated at 37 °C in the presence of the anionic cofactor heparin at a TauRD:heparin molar ratio of 8:1. The aggregation was allowed to proceed for 3 days.

We hypothesized that vaccination-induced antibodies that specifically recognize pathological tau conformers would prevent and resolve PHF-mediated tauopathies with high efficacy and safety. To generate an appropriate tau conformer antigen in the PHF configuration, we selected TauRD (2N4R-hTau amino acid 243–368) (Fig. 1a). TauRD plays a crucial role in hyperphosphorylation-induced PHF formation^7^. We manufactured the TauRD protein by using the pTYB12 plasmid system (Fig. 1b) and then tested whether the recombinant TauRD protein could induce PHF and NFT-resembling tangle formation. The recombinant TauRD migrated as a 14 kDa band on SDS-PAGE, and this band was detected by Western blot analysis using the A-10 antibody (Fig. 1c). Because NFTs constitute a quaternary (4D) protein structure, we employed nondenaturing native PAGE to analyze the aggregation status of TauRD. Previously, we reported that native PAGE can be used to reveal the quaternary conformations of proteins^60^. On the other hand, SDS-PAGE can dissociate TauRD aggregates into monomer polypeptide chains. Thus, the NFT-resembling TauRD aggregates and monomers can be characterized by analysis of the TauRD polypeptide via native PAGE and SDS-PAGE, respectively. Given that tau PHFs adopt a β-sheet conformation and that thioflavin S (ThS) intercalates with the β-sheets, we observed whether the β-sheet PHF conformation was maintained in native PAGE by staining with ThS. Briefly, we incubated freshly purified or heparin-treated PHF TauRD with ThS and carried out native PAGE. To prepare heparin-treated TauRD, we treated the TauRD proteins (in PBS) with heparin at a 1:8 ratio (heparin:TauRD) for 5 days at 37 °C without shaking. As shown in Fig. 1d, the majority of the TauRD aggregates remained in the loading well in the presence of heparin or near the interface between the stacking and running gels in the absence of heparin. After staining with ThS, the heparin-treated TauRD aggregates exhibited granular bright green fluorescence, which indicated that the aggregates consisted of β-sheets (Fig. 1e). Heparinization of TauRD revealed a unique ultrastructure of TauRD aggregation (Fig. 1f). TauRD also forms NFT tangle-like structures (Supplementary Fig. 1). This result strongly suggested that the recombinant TauRD polypeptide aggregates into conformers with stable β-sheet structures, which resemble the NFTs observed in tauopathy neuronal cells. A suspension of TauRD in PBS was used for further animal immunization studies.

### Induction of TauRD-specific antibody responses by intranasal immunization in combination with the FlaB adjuvant

**Fig. 2:**
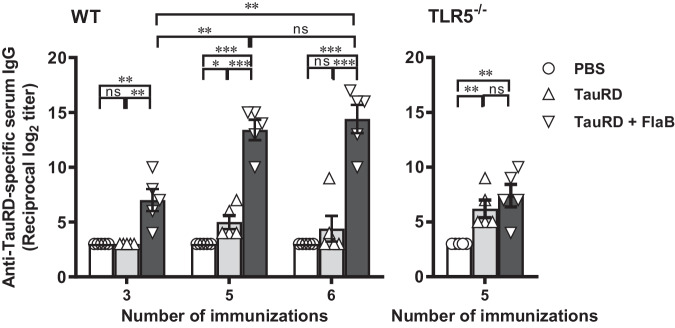
Antigen-specific antibody response following intranasal immunization with FlaB-adjuvanted TauRD. Wild-type BALB/c or TLR5 KO (BALB/c background) mice were subjected to intranasal administration of one of the following at one-week intervals: PBS, PBS containing 10 µg of TauRD, or PBS containing 10 µg of TauRD and 4 µg of FlaB (TauRD+FlaB). Following the third, fifth, and sixth rounds of immunization, the levels of TauRD-specific serum IgG were evaluated using an enzyme-linked immunosorbent assay (ELISA). Statistical differences were analyzed by Student’s *t* test for unpaired means. *P < 0.05, **P < 0.01, ***P < 0.001, ^ns^ not significant. The error bars represent the standard error of the mean (SEM) values.

We initially optimized the immunization schedule to provide strong TauRD-specific antibody responses. Mice received intranasal immunizations consisting of 10 µg of TauRD and 4 µg of FlaB. To assess TauRD-specific antibody titers, serum samples obtained after three, five, and six rounds of immunization were analyzed using an enzyme-linked immunosorbent assay (ELISA). As shown in Fig. 2, immunization with TauRD+FlaB induced significantly higher IgG antibody responses than did immunization with TauRD alone (3 rounds, TauRD vs. TauRD+FlaB **P < 0.01; both 5 and 6 rounds, TauRD vs. TauRD+FlaB ** *P* < 0.001). TauRD alone failed to induce a notable antibody response, in contrast to the group receiving the TauRD+FlaB. Five and six rounds of immunization with TauRD+FlaB induced comparable antibody responses (*P* > 0.05), suggesting that the ceiling of the immune response was achieved by five rounds of immunization.

Previous studies demonstrated the direct binding of FlaB to human TLR5, leading to the activation of NF-κB^54,60^. To confirm whether immunopotentiation effect of FlaB on TauRD was mediated by TLR5 signaling, we conducted a comparative analysis between BALB/c wild-type (WT) and TLR5^−/−^ mice. The mice were vaccinated five times with 10 µg of TauRD with or without 4 µg of FlaB, at one-week intervals. The PBS and TauRD-only groups served as controls. In contrast to the result in WT mice, the immunopotentiation effect was completely abrogated in TLR5^-/-^ mice (Fig. 2). This result confirmed that the immune enhancement of TauRD-specific immune responses by FlaB is indeed mediated by the host TLR5 signaling pathway. To investigate vaccine-induced Th1 vs Th2 responses, we characterized the IgG isotypes of anti-TauRD serum antibodies in five-time immunized mice. IgG2a isotype was significantly induced in FlaB+TauRD immunized mice but the level was lower than that of IgG1. We calculated the TauRD-specific serum IgG2a/IgG1 ratio after five times immunization. The IgG2a/IgG1 ratio in TauRD or FlaB+TauRD immunized mice was 0.870 ± 0.083 or 0.698 ± 0.041, respectively (Supplementary Fig. 2a). These results indicate that the immune balance induced by FlaB-adjuvanted TauRD was biased towards Th2-mediated humoral immune responses.

TauRD includes four KXGS motifs that regulate microtubule affinity and aggregation kinetics. It has been reported that tau hyperphosphorylation is the primary catalyst that initiates autoassembly into PHFs, which eventually polymerize to form NFTs^65^. Moreover, pseudorepeats contribute to the formation of β-sheet structures in pathological tau NFTs. Those conserved KXGS motifs are located at the core region of tau protofilaments, which pack each other to form PHFs^66^. In this context, we formulated a vaccine composed of these pseudorepeat peptides as a control antigen. We employed peptide antigens consisting of Tau_sp_1 (^253^LKNVKSKIGS^262^), Tau_sp_2 (^284^LSNVQSKCGS^293^), Tau_sp_3 (^319^TSKCGSLGNI^328^), and Tau_sp_4 (^347^KDRVQSKIGS^356^) (the conserved motif sequence is underlined; see Supplementary Table 1). Mice were intranasally immunized with a mixture of the four short peptides (Tau_sp_mix; 25 µg each) alone or in combination with 4 µg FlaB (Tau_sp_mix+FlaB), after which the TauRD-specific Ab responses were evaluated. Although the antibody level remained negligible after the third vaccination, the addition of FlaB to Tau_sp_mix significantly increased TauRD-specific IgG antibody production after five vaccinations (*** *P* < 0.001). Notably, the log_2_ IgG titers elicited by five immunizations with Tau_sp_mix+FlaB were significantly lower than those induced by TauRD+FlaB (7.40 ± 0.68 vs. 13.40 ± 0.93, *** *P* < 0.001), despite the substantially greater total quantity of Tau_sp_mix administered (100 µg) than that of TauRD (10 µg) (Supplementary Fig. 2b).

### Specific recognition of TauRD conformers by anti-TauRD+FlaB sera

**Fig. 3:**
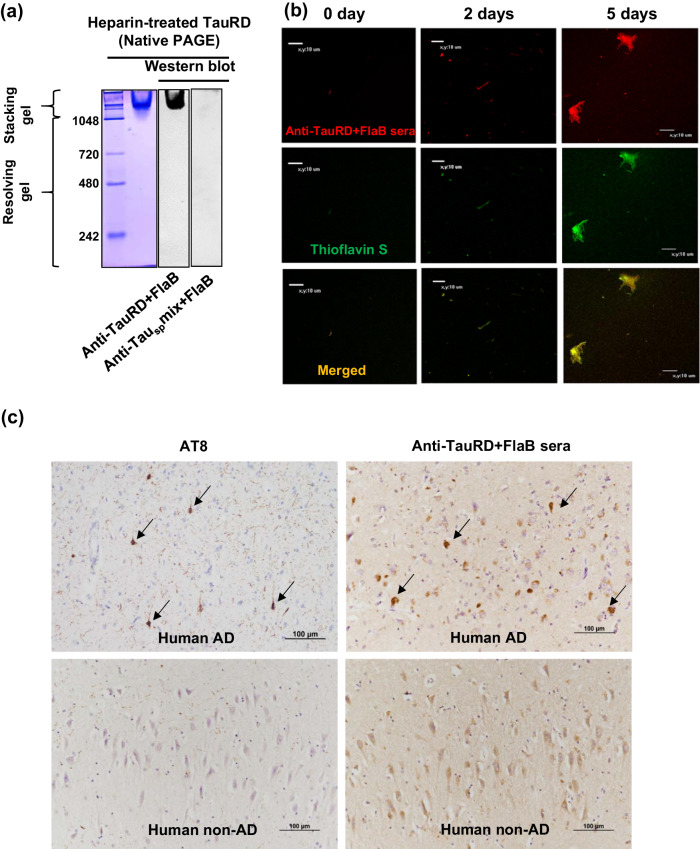
Characterization of anti-TauRD+FlaB sera induced by repeated intranasal immunization. **a** Immunoblot analysis of heparin-treated TauRD aggregates separated by native PAGE using anti-TauRD+FlaB sera induced by repeated intranasal immunization. Heparinized TauRD proteins were resolved using native-PAGE, and subsequently, the TauRD protein bands were detected and visualized using antiserum induced by intranasal immunization with TauRD and FlaB (1:500 dilution). Postimmune serum obtained from the Tau_sp_mix+FlaB group was used as an antibody with sequence-specific recognition of tau protein at a 1:200 dilution. **b** Immunofluorescence detection of heparin-treated TauRD aggregates using anti-TauRD+FlaB sera from mice that received intranasal immunization. Heparin-treated TauRD fibrils were double stained with thioflavin S and postimmune serum from the FlaB+TauRD group. **c** Immunohistochemical detection of human Alzheimer’s disease tauopathy specimen using anti-TauRD+FlaB serum from mice that received intranasal immunization. Immunohistochemistry of anti-TauRD+FlaB serum revealed strong and specific staining in the neurofibrillary tangles of the hippocampi of humans with Alzheimer’s disease (black arrows). The staining results were compared with the IHC result obtained with phospho-PHF-tau pSer202+The205 antibody (AT8; Invitrogen) from the same patient with Alzheimer’s disease.

Next, we characterized the antiserum obtained from mice immunized with TauRD+FlaB. The recombinant TauRD protein was separated via SDS-PAGE and subsequently transferred onto membranes for Western blot analysis. Because Tau_sp_mix is a peptide cocktail, it is conceivable that this antigen induces antibodies with a specific affinity for amino acid sequences rather than for the pathological PHF conformation. Consequently, we utilized anti-Tau_sp_mix+FlaB serum as a sequence-specific control. On SDS-PAGE, the TauRD aggregates almost completely dissociated into monomers, as evidenced by Ponceau S staining. The TauRD monomer band was distinctively detected in the anti-Tau_sp_mix+FlaB serum sample. On the other hand, intriguingly, the anti-TauRD+FlaB serum sample exhibited a negligible monomer band but displayed strong reactivity toward a higher-molecular-weight band that was not detected by Ponceau S (Supplementary Fig. 3). To confirm whether anti-TauRD+FlaB specifically recognized fibrillized TauRD, we prepared heparin-treated TauRD and performed native PAGE followed by Western blot analysis. As shown in Fig. 3a, the anti-TauRD+FlaB antibodies reacted strongly to aggregated TauRD within the stacking gel, whereas the anti-Tau_sp_mix+FlaB antibodies did not. Considering that Tau_sp_mix is a cocktail of short peptides, this antigen mixture should induce antibodies specific to amino acid sequences, which should be hidden when pathological conformers are formed and should be unable to recognize conformational structures. These findings suggest that mice immunized with TauRD+FlaB primarily should exhibit specific antibody responses against pathological tau conformers.

To further verify whether the anti-TauRD+FlaB serum has an affinity for PHFs, we induced fibrillization of recombinant TauRD in the presence of heparin at 37 °C and harvested the fibrillized proteins on Days 2 and 5 for double staining with ThS and immunofluorescence antibodies. On Day 2, the TauRD protein underwent aggregation, forming PHFs that exhibited specific binding to the anti-TauRD+FlaB serum. This evident binding with intense fluorescence exclusively colocalized with the ThS signals. By Day 5, the TauRD fibrillary aggregation had further developed into dendritic tangles, which exhibited stronger staining with both ThS and antibodies (Fig. 3b). To determine whether the conformer-specific antibodies present in the anti-TauRD+FlaB serum could recognize PHFs in human tauopathy lesions, we conducted immunohistochemistry (IHC) on human AD brain specimens. As a positive control, we used an anti-phospho-PHF-tau pSer202+The205 (AT8) antibody. The conformer-specific antibodies induced by TauRD+FlaB immunization exhibited the ability to reveal intracytoplasmic neurofibrillary tangles in the human hippocampus with AD neuropathologic change. The staining specificity was comparable to that of the commercially available pathological tau-specific AT8 antibody (Fig. 3c).

### Inhibition of PHF assembly by conformer-specific anti-TauRD+FlaB serum

**Fig. 4:**
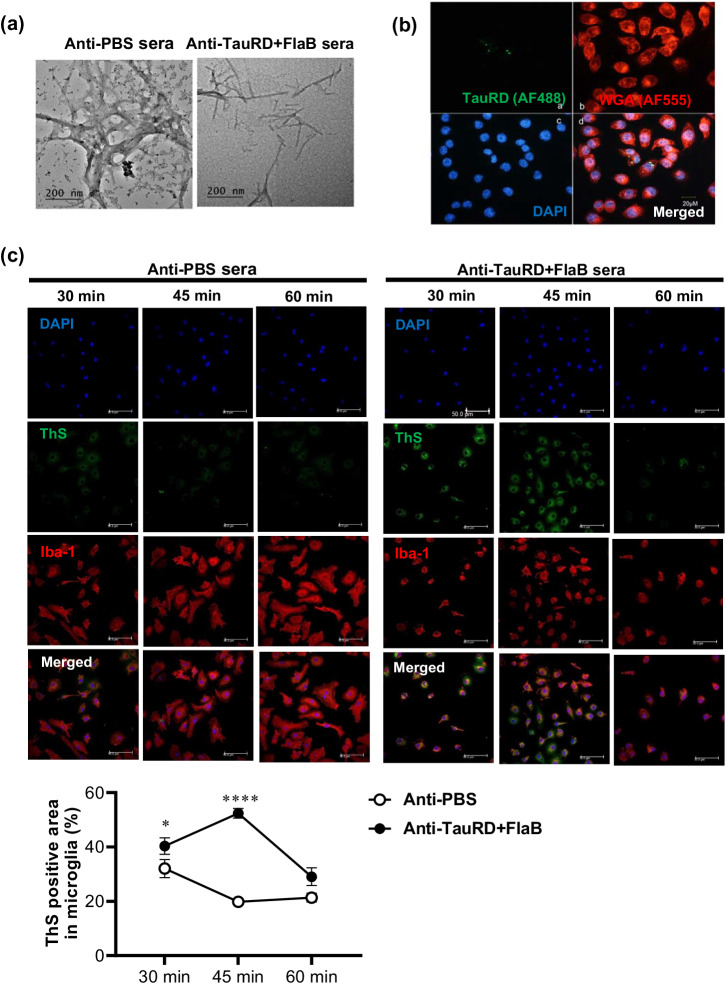
Functional characterization of anti-TauRD+FlaB serum from mice received repeated intranasal immunization. **a** TEM analysis of TauRD aggregation in the presence of anti-TauRD+FlaB serum from mice that receive intranasal immunization. TauRD protein was pretreated with either anti-PBS or anti-TauRD+FlaB serum and then induced to aggregate for 5 days in the presence of heparin. Fluorescence micrographs (**b**) demonstrating the uptake of TauRD aggregates by BV2 cells. Heparin-treated TauRD fibrils were first sonicated, then incubated with anti-TauRD+FlaB serum. Subsequently, BV2 cells were treated with the TauRD-antiserum complex. Uptake of TauRD aggregates by BV2 cells were observed using wheat germ agglutinin stain (WGA). **c** Representative images show that TauRD uptake in the primary cultured microglia. Primary mouse microglial cells were prepared and then the cells were treated with anti-PBS or anti-TauRD+FlaB sera. TauRD uptake was quantified by ThS positive area in Iba-1^+^ microglia and analyzed by two-way ANOVA analysis followed by Tukey’s multiple comparisons test. *P < 0.05; **** *P* < 0.0001 compared with the PBS (control).

Subsequently, we evaluated whether the conformer-specific polyclonal antibodies induced by multiple intranasal immunizations with TauRD+FlaB could inhibit TauRD tangle formation. IgG antibodies were purified from the sera of mice intranasally immunized with PBS (control) or TauRD+FlaB. The purified IgG antibodies were then incubated with the TauRD protein at 4 °C overnight with end-over-end rotation. Subsequently, the TauRD proteins were incubated with the purified IgG antibodies and subjected to aggregation for 5 days in the presence of heparin at 37 °C, after which the morphology of the TauRD aggregates was examined via TEM. While TauRD proteins treated with control antibodies exhibited compact dendritic fibrillar tangles, tangle formation was significantly inhibited in the presence of TauRD+FlaB IgG antibodies (Fig. 4a). This result indicates that the conformer-specific antibodies present in the anti-TauRD+FlaB sera significantly reduced the formation of dense tangles, potentially leading to the dispersion of PHFs into shorter and thinner filaments, which could be more readily engulfed by microglia.

### Conformer-specific antibodies facilitated the uptake of TauRD oligomeric aggregates by microglia

Microglia, the resident innate phagocytic cells of the brain, efficiently clean antibody-bound tau oligomers from the extracellular space^67^. It has been reported that therapeutic tau antibodies can enhance the internalization of tau aggregates by microglia^68^. Hence, we investigated whether conformer-specific antibodies targeting TauRD aggregates could facilitate the in vitro phagocytic degradation of TauRD aggregates by microglial cells. Initially, we subjected heparin-treated TauRD fibrils to sonication to fragment the TauRD tangles into smaller segments. Subsequently, we incubated BV2 cells with these TauRD tangle fragments in the presence of anti-TauRD+FlaB serum, and assessed phagocytosis. After 30 min of incubation, TauRD tangle fragments were evidently observed inside of BV2 cells (Fig. 4b).

We further assessed microglial cell-mediated phagocytosis of TauRD tangle fragments by using mouse primary microglia. As shown in Fig. 4c, conformer-specific anti-TauRD+FlaB serum significantly enhanced the internalization of TauRD tangle fragments by primary microglia compared to the treatment with anti-PBS serum. Interestingly, the phagocytosis efficiency of primary microglia was significantly greater than that of BV2 cells, and the intensity of the intracellular ThS stain decreased over time, suggesting active degradation of the TauRD aggregates. These results suggested that the pathological Tau conformer-recognizing antibodies produced by TauRD+FlaB immunization would also induce the efficacious clearance of extracellular tau aggregates by microglia in vivo.

### Development of a built-in adjuvanted FlaB-TauRD fusion anti-tauopathy vaccine

**Fig. 5:**
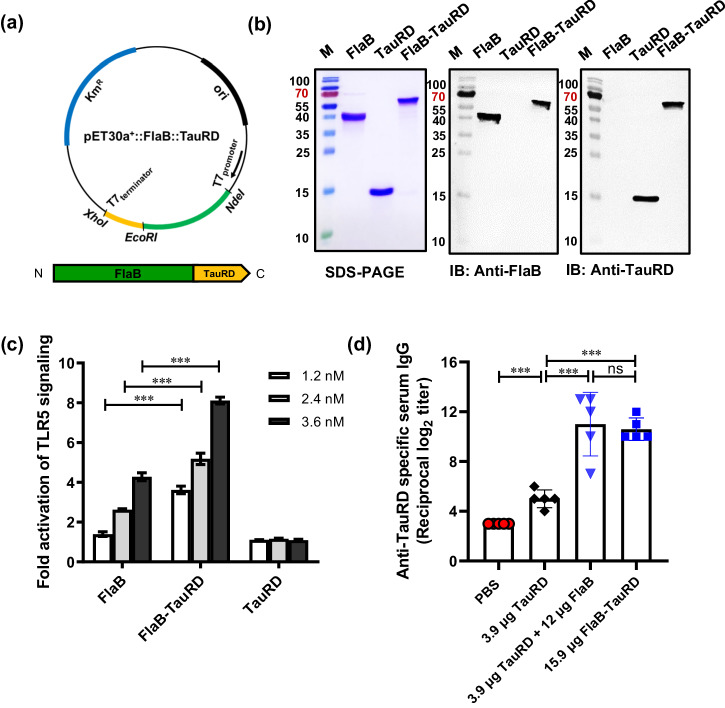
Generation and characterization of the built-in adjuvanted FlaB-TauRD vaccine. **a** Construction of the expression vector for the built-in adjuvanted FlaB-TauRD vaccine. DNA fragments of Vv-flaB, TauRD, the T7 promoter region, a kanamycin resistance gene (Km), and cloning restriction enzyme sites are shown. **b** SDS PAGE and Western blot analysis of the purified recombinant FlaB-TauRD fusion protein. The FlaB-TauRD protein was probed with an anti-FlaB or anti-TauRD antibody whose production was elicited by the intraperitoneal injection of FlaB or TauRD in combination with Freund’s adjuvant. **c** Determination of TLR5-dependent NF-κB-stimulating activity by FlaB-TauRD. The relative luciferase activity levels in the cell extracts were analyzed by a dual-luciferase reporter assay system and normalized to that of the pCMV-β-galactosidase plasmid as a control. The same molar ratios of proteins were used, and PBS was used as a negative control. **d** Comparison of TauRD-specific Ab titers between anti-TauRD+FlaB and anti-FlaB-TauRD sera. Statistical differences were analyzed by Student’s *t* test for unpaired means. *P < 0.05, **P < 0.01, ***P < 0.001, ^ns^ not significant. The error bars represent the standard error of the mean (SEM) values.

Next, to assess the amelioration of tauopathy progression in vivo by intranasal vaccination, we constructed a built-in adjuvanted vaccine comprising FlaB and TauRD (Fig. 5a). A recombinant built-in adjuvanted vaccine, in which the TauRD antigen and FlaB adjuvant are fused into a single polypeptide, would present greater translational potential as a pharmaceutical product. The recombinant FlaB-TauRD fusion protein exhibited a single band with a molecular mass of 57 kDa in SDS-PAGE. This band was subsequently confirmed by Western blot analysis using both anti-FlaB and anti-tau (A-10) (Fig. 5b). To confirm the correct folding and functionality of the built-in adjuvanted vaccine, we checked the TLR5-stimulating activity of the FlaB-TauRD fusion protein and observed dose-dependent TLR5-stimulating activity (Fig. 5c). Notably, at the same stoichiometry, the TLR-5-stimulating activity was greater than that of FlaB, indicating that the fusion partner protein does not impede TLR5-binding activity and may expose even more TLR5-binding motifs.

To compare the TauRD-specific antibody responses elicited by both the mixture and built-in adjuvanted anti-tauopathy vaccines with equimolar ratios of TauRD and FlaB, we measured TauRD-specific IgG titers in the serum. BALB/c mice received intranasal vaccination with 3.9 μg of TauRD, 3.9 μg of TauRD plus 12 μg of FlaB, or 15.9 μg of the FlaB-TauRD fusion protein five times at one-week intervals. The results revealed comparable levels of TauRD-specific serum IgG titers between the built-in adjuvanted (FlaB-TauRD) and mixed (TauRD+FlaB) vaccine formulations (Fig. 5d). We confirmed that FlaB-TauRD fusion protein also forms fibrillar structure similar to TauRD by transmission electron microscopy (TEM) and induces the conformation-specific antibodies by native-PAGE, and Western blot analysis after SDS-PAGE (Supplementary Fig. 5a–c). These findings encouraged us to proceed to the preclinical trial of the FlaB-TauRD fusion vaccine in a transgenic animal model.

### Amelioration of tauopathy in P301S transgenic mice by mucosal immunization with the FlaB-TauRD vaccine

To assess the potential of the FlaB-TauRD fusion vaccine for clinical application, we vaccinated P301S human tau transgenic mice and observed the effect of vaccination on disease progression. In our animal facility, behavioral symptoms related to tauopathy, such as clasping and limb retraction, began to manifest approximately three months after birth. As the tauopathy progressed, we observed worsening manifestations such as limb weakness associated with hunched-back posture, and debilitating paralysis. We assessed the presence of pathologic tau accumulation in the brain tissues of P301S mice by immunohistochemical analysis employing an antibody specific for PHF-tau phosphorylated at Ser202 and Thr205 (AT8). As illustrated in Supplementary Fig. 4, tauopathy progressively developed in the P301S transgenic mice over the observation period.

**Fig. 6:**
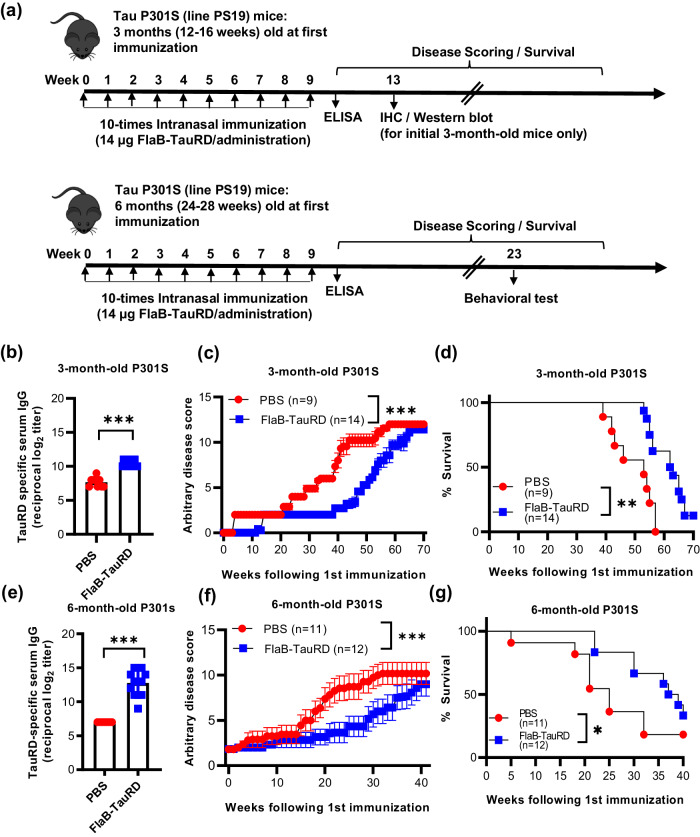
Amelioration of tauopathy in P301S-transgenic mice through active immunization with the built-in adjuvanted FlaB-TauRD vaccine. **a** Experimental schedule for multiple immunizations with the recombinant FlaB-TauRD fusion protein. At the age of either three or six months, P301S mice were intranasally immunized with either phosphate-buffered saline (PBS) or 14 μg of FlaB-TauRD at weekly intervals for a total of ten times. **b**, **e** TauRD-specific serum IgG levels following ten rounds of intranasal immunization with FlaB-adjuvanted TauRD. One week after the final immunization, levels of TauRD-specific serum IgG were evaluated using an enzyme-linked immunosorbent assay (ELISA). **c**, **f** Determination of the disease score of tauopathy in P301S-transgenic mice. **d**, **g** Measurement of the survival of vaccinated mice. Following multiple rounds of immunization, the disease score and survival of the immunized P301S mice were assessed. Experimental schedule of the behavioral tests.

To investigate the efficacy of the anti-tauopathy protective immune responses elicited by active immunization, 3- or 6-month-old P301S transgenic mice were intranasally immunized with either PBS or 14 μg of FlaB-TauRD every week. TauRD-specific antibody responses and tauopathy progression were assessed over the time course (Fig. 6a). First, to test whether FlaB-generated immune responses are contributing to tauopathy disease protection regardless of fusion to the TauRD, we employed FlaB alone immunization group. As shown in Supplementary Fig. 5d, there was no significant difference between FlaB alone and PBS groups in terms of disease severity and survival. This finding reveals that FlaB alone does not significantly influence tauopathy progression in P301S mice.

As depicted in Fig. 6b, e, both 3- and 6-month-old P301S mice subjected to 10 rounds of FlaB-TauRD immunization demonstrated significantly higher TauRD-specific antibodies than the PBS control group (***P < 0.001). Notably, PBS-immunized P301S mice (Fig. 6b, e) had significantly higher background anti-TauRD antibody titers than those in isogenic WT mice (Fig. 5d), suggesting that endogenous transgenic P301S tau induces notable antibody responses over time.

To assess whether the anti- FlaB-TauRD antibodies raised in P301S mice effectively inhibit tau aggregation, we conducted the above-mentioned in vitro aggregation inhibition assay. As depicted in the Supplementary Video, the anti-FlaB-TauRD serum efficaciously inhibited tau aggregate formation. The anti-PBS serum, which also exhibited a substantial anti-TauRD antibody titer, did not exhibit any discernable inhibition of TauRD aggregate formation. After 96 h of incubation, robust tau aggregate formation was observed in samples treated with anti-PBS, whereas aggregates were not observed in samples treated with anti-FlaB-TauRD. These findings demonstrated that FlaB-TauRD vaccination induced the production of functionally active antibodies in P301S mice also.

To determine whether active immunization elicited significant phenotypic improvements in P301S mice afflicted by tauopathy, we monitored disease scores and survival as long as 70 or 40 weeks after the start of vaccination. Both 3- and 6-month-old P301S mice that received ten FlaB-TauRD immunizations exhibited significantly delayed onset of the signs and symptoms of tauopathy such as hind leg clasping, hunched back, weakened hind leg resulting in impaired forward movements, and paralysis (***P < 0.001, PBS vs. FlaB-TauRD for both age groups) (Fig. 6c, f). In our animal facility, tauopathy phenotypes started to be observed after 3 months of age. We have started vaccination in 6-month-old P301S mice since we wanted to see whether FlaB-TauRD would be effective when tauopathy pathology substantially progressed. Remarkably, FlaB-TauRD-immunized P301S mice exhibited significantly longer survival than did their PBS-immunized counterparts in both the 3-month-old and 6-month-old P301S mouse groups (**P < 0.01, PBS vs. FlaB-TauRD in 3-month-old mice; *P < 0.05, PBS vs. FlaB-TauRD in 6-month-old mice) (Fig. 6d, g). An earlier start (3 months) of vaccination resulted in a significantly later onset of tauopathy symptoms and longer survival, although the induced antibody levels were lower than those in mice started immunization after 6 months (Fig. 6b–g).

**Fig. 7:**
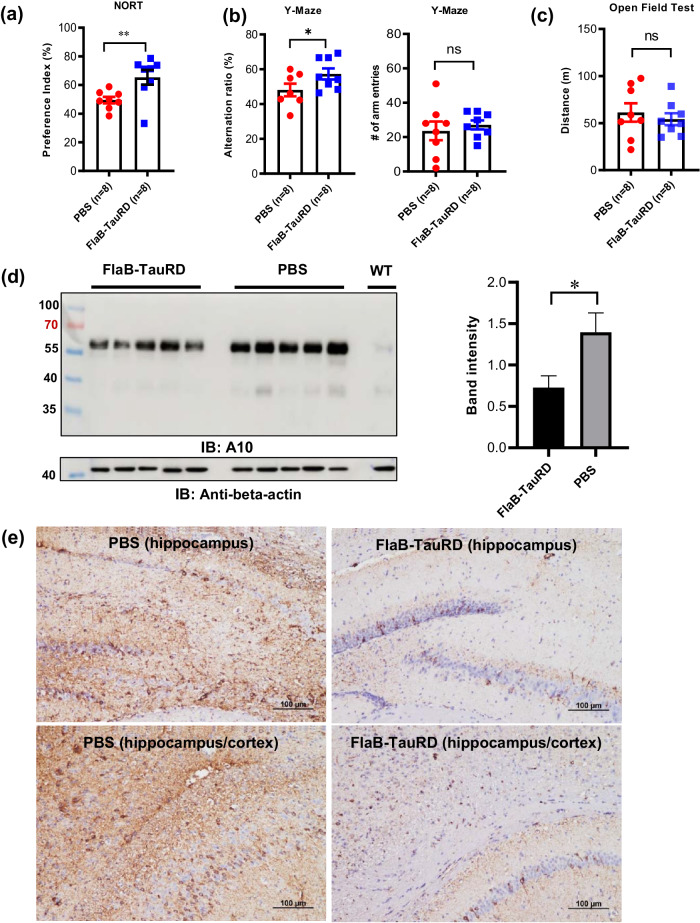
Improvement of behavioral traits of P301S-transgenic mice through active immunization with the built-in adjuvanted FlaB-TauRD vaccine. Fourteen weeks after the tenth immunization, the novel objective recognition test (NORT) (**a**), Y-maze test (**b**), and the open field test (**c**) were carried out. **d** Determination of tau protein in the P301S mouse brain. Western blot analysis was conducted to assess tau protein levels in the P301S mouse brain. Each well was loaded with 2 micrograms of brain protein. **e** Determination of hyperphosphorylated tau in the P301S mouse brain. Immunohistochemical analysis was performed to examine hyperphosphorylated tau using the AT8 antibody in P301S transgenic mice. The analysis included the hippocampus and cerebral cortex of both PBS- and FlaB-TauRD-vaccinated P301S transgenic mice.

To evaluate the impact of FlaB-TauRD vaccination on the behavioral traits of 6-month-old P301S mice (average disease score 1.0 ± 0.0) (Supplementary Fig. 5e), we conducted a novel objective recognition test (NORT) (Fig. 7a), Y-maze tests (Fig. 7b), and open field test (Fig. 7c) 14 weeks after the last vaccination. Age-matched mice that received PBS vaccinations (disease score 8.5 ± 1.30) were evaluated as non-vaccination controls (Supplementary Fig. 5e). The NORT evaluates recognition memory, and the Y-maze tests assess reference memory and working memory^69^. In the NORT, the PBS-immunized group allocated a comparable amount of time to both objects (the same object and novel object). In contrast, the FlaB-TauRD-immunized group spent more time inspecting the novel object than the familiar object, resulting in a higher preference index, indicating enhanced recognition memory (**P < 0.01, PBS vs. FlaB-TauRD) (Fig. 7a). In the Y-maze test, which assesses the impairment of working memory, the FlaB-TauRD-immunized group displayed a significantly improved alternation ratio, indicating enhanced working memory (*P < 0.05, PBS vs. FlaB-TauRD) (Fig. 7b). Notably, there were no discernible differences in the total number of arm entries between the two groups, implying similar motor function in both groups at the tested ages (Fig. 7b). The open field test revealed comparable basal locomotor activity in both the PBS- and FlaB-TauRD-immunized groups (Fig. 7c), suggesting that FlaB-TauRD immunization did not affect general motor activity in these two groups.

To investigate underlying mechanism of immunization-mediated protective effect, we carried out the biochemical and immunohistochemical analyses of pathologic tau in the brains of vaccinated P301S transgenic mice. As shown in Fig. 7d, while age-matched wild-type mice did not manifest pathological tau accumulation in the brain, P301S TG mice had significantly accumulated pathologic tau proteins in the brain. Notably, FlaB-TauRD-vaccinated mice showed significantly decreased levels of Tau proteins in the brain (*P* < 0.05). In addition, we conducted IHC for pathological tau in vaccinated P301S TG mouse brains. We stained pathological tau in the brain of mice immunized ten times with FlaB-TauRD with the AT8 antibody that specifically binds to phosphorylated tau. As shown in Fig. 7e, the accumulation of pathological Tau in the brain of vaccinees dramatically decreased as compared to age-matched PBS control. These results suggest that active immunization of FlaB-TauRD fusion protein significantly lowered the accumulation of pathological Tau in vivo.

This outcome demonstrated that active immunization with the FlaB-TauRD vaccine engenders a protective immune response in P301S-transgenic mice, leading to improved quality of life and extended survival. In summary, these findings collectively provide compelling evidence that active immunization with the built-in adjuvanted FlaB-TauRD vaccine effectively mitigates tauopathy, ameliorating both clinical and pathological aspects of the disease.

## Discussion

Immunotherapy for tauopathies should selectively target pathological tau molecules while preserving physiologically functioning tau molecules. Immunological effectors should selectively prevent further PHF tangle formation and mediate the clearance of already-formed tau aggregates^33^. Preventing the transcytosis of prion-like pathological tau seeds released from dying neuronal cells is also important for improving therapeutic efficacy^2^. In the present study, we generated a recombinant TauRD polypeptide that is prone to PHF formation and tested its effectiveness as an antitauopathy vaccine antigen in combination with the mucosal adjuvant FlaB. Notably, the antibodies induced by vaccination predominantly targeted the PHF conformers of TauRD. These conformer-specific antibodies effectively suppressed the aggregation of TauRD into tangles and facilitated the phagocytic degradation of pre-existing tangle fragments. Based upon these in vitro observations, we developed a built-in adjuvanted FlaB-TauRD fusion polypeptide as an antitauopathy vaccine and confirmed that active immunization with the FlaB-TauRD vaccine ameliorated tauopathy in a P301S human tau transgenic mouse model. This is, to our knowledge, the first report of the efficacious induction of antibody responses specifically targeting pathological tau conformers and the therapeutic efficacy of TauRD PHF conformer-targeting active immunization through the intranasal route.

Tau proteins contain numerous charged residues: the net charge of 4R-TauRD at neutral pH is predicted to be +10, and its isoelectric point (pI) is 10.46. Polyanions such as heparin, RNA, and polyglutamate, which compensate for the positive charges of aggregates, have been reported to increase the efficiency of TauRD assembly much faster than that of full-length tau protein^42,70–72^. Even in the absence of polyanions, the recombinant TauRD polypeptide purified by the IMPACT-CN System (New England Biolabs) showed a high propensity for aggregation, as revealed by nondenaturing native PAGE and ThS staining (Fig. 1d). Fresh recombinant TauRD protein monomers could not be separated by native PAGE and assembled as granular structures that were readily stained by ThS, indicating the formation of a rather regular β-sheet structure in buffered solutions. In contrast to proteins obtained by using other purification systems, proteins expressed by the pTYB12 vector and purified by the IMPACT-CN System were subjected to DTT-induced on-column cleavage for 48 h at room temperature. Given that tau assembly can be promoted by elevated temperature in a time-dependent manner^65,72^, our protein purification procedure might have contributed to the formation of TauRD oligomers and aggregates.

A recent study involving epitope analysis of the tau protein predicted low immunogenicity of TauRD and revealed five immunogenic motifs within the N-terminal, proline-rich, and C-terminal regions of the tau protein^73^. We agree to some extent that TauRD has low immunogenicity since short peptide antigens or TauRD alone did not induce meaningful antibody responses even after six rounds of immunization at relatively high doses (Fig. 2 and Supplementary Fig. 2b). Moreover, AADvac1 uses the keyhole limpet hemocyanin-conjugated ^294^KDNIKHVPGGGS^305^ peptide in TauRD as a vaccine epitope, which might have contributed to the generation of significant antibody responses in animal and human subjects^46,47^. For the short unique peptide antigens in the repeat region (Tau_sp_mix) used in this study, the average antibody titer after 6 rounds of adjuvanted immunization was below 10log_2_, which was rather low to have substantial in vivo efficacy. On the other hand, three rounds of immunization with hypoimmunogenic TauRD, adjuvanted with FlaB-induced antibody titers similar to those after 6 rounds of immunization with Tau_sp_mix+FlaB. After 5 rounds of immunization, the TauRD+FlaB group reached antibody titers 15log_2_ in a TLR5-dependent manner (Fig. 2). Remarkably, the serum generated from anti-TauRD+FlaB immunization predominantly recognized PHF tangle conformers (Fig. 3). Compared with previously studied microbial antigens, the TauRD antigen, which is speculated to be relatively hypoimmunogenic, requires a higher dose and more repeated immunizations even when combined with flagellin^54,58^. These results suggest that for clinical application, the TauRD+FlaB vaccine should be administered regularly for a long period to maintain effective antibody titers. The immunization interval also had a significant influence on the resulting immune responses. The 2-week interval was not as effective as the 1-week interval (data not shown). This result indicates that more frequent administration and higher concentrations of the hypoimmunogenic TauRD antigen are needed for effective humoral immune response to generate conformer-specific antibodies. Moreover, the kinetics of antibody induction after vaccination were evaluated. Ten micrograms of the TauRD vaccine formulated with FlaB required five rounds of immunization to reach the ceiling of Ab responses, although significant responses were noted after three rounds (Fig. 2). Given the pharmacokinetic advantages of IgG Abs and the low immunogenicity of the TauRD antigen, a vaccination strategy with more rounds and more frequent administration will be needed in future human trials to secure isotype switching to IgG and maintain therapeutic levels. Compared with other adjuvanticity mechanisms, TLR5 signaling through the mucosal compartment likely provides a unique advantage in potentiating immune responses against TauRD epitopes. We observed that subcutaneous administration of the same dose of the short peptide mixture with complete Freund’s adjuvant (CFA) failed to stimulate antibody production (data not shown). CFA, a mixture of strong ligands for pattern recognition receptors on antigen-presenting cells that is regarded as the strongest experimental adjuvant, failed to induce immune responses against short-peptide TauRD antigens, while FlaB induced substantial antibody responses after only three rounds of immunization. Flagellin is also a cognate ligand of the NLRC4 inflammasome pathway^59,74^. Nevertheless, in the present study, NLRC4 is not believed to play a dominant role in enhancing the immune response, given that the adjuvant effect was completely eliminated in TLR5-KO mice. Along with cytosolic antigen processing, NLRC4 activation induces robust cytotoxic T lymphocyte responses^59^. A recent report noted that microglia-mediated T-cell infiltration aggravated neurodegeneration in tauopathy^75^. In this regard, an antitauopathy vaccine should direct the immune balance toward humoral immunity. To this end, the NLRC4 binding domain could be deleted in the development of a clinical-grade FlaB-TauRD vaccine.

An effective and safe Tau-based AD vaccine must have a high benefit/risk ratio for administration to a relatively aged population that might already exhibit the beginning of neuronal degeneration. Such a vaccine must induce an adequate antibody response against pathological tau molecules while restraining adverse responses to physiological ones. Notably, antibodies generated in response to the FlaB-adjuvanted TauRD vaccine selectively recognized the multimeric form of TauRD (Fig. 3a and Supplementary Fig. 3). As reported, the immunization of wild-type mice with full-length tau recombinant protein in combination with CFA and pertussis toxin (PT) leads to tauopathy-like abnormalities and neurological deficits^17^, which are presumably related to the use of full-length human tau protein differing from the mouse sequence or the use of strong adjuvants such as CFA and PT that induce a strong cytotoxic T-cell response. The mucosal immune system has evolved a variety of mechanisms to achieve and maintain the tolerance of self-antigens, and the degree of tolerance relies on the dose, route, and frequency of autoantigen administration^49^. Thus, a small proportion of monomeric TauRD administered intranasally might have been processed as tolerogenic self-antigens, while the TauRD PHF conformers were recognized as foreign antigens. However, a high dose of a short-peptide mixture formulated with FlaB also induced the production of serum antibodies that recognized only the monomeric form of TauRD. In this case, a high dose of the short-peptide mixture likely overwhelmed the tolerance of the mucosal immune system with the help of the mucosal adjuvant FlaB. Accordingly, mucosal vaccination with TauRD formulated with the FlaB adjuvant likely has advantages over systemic administration in terms of safety. Nevertheless, the immunogenicity of FlaB could present concerns. Repeated administration of these agents inevitably induces the production of anti-FlaB antibodies, which may nullify the TLR5-FlaB interaction and might lead to hypersensitivity. To address this problem, we generated deimmunized FlaB by deleting the dominant B-cell epitope^60,76^.

## Methods

### Construction and purification of recombinant TauRD and FlaB-TauRD proteins

To generate recombinant TauRD protein, the DNA sequence encoding the 4R-tau repeat region (amino acids 243-368) from 2N4R-human tau was synthesized by Bioneer (Daejeon, Korea) (Fig. 1a). To produce a recombinant FlaB-TauRD fusion protein, we generated a pET30a+ plasmid (Novagen) containing a DNA fragment encoding TauRD (amino acids 243-368) (Table 1). The inserted DNA fragment was generated through PCR amplification, utilizing a codon-optimized DNA template and the following primer pair: tauRD-F and tauRD-R, with EcoRI and XhoI sites, respectively (Table 2). The codon usage was systematically optimized to increase the expression of the resulting gene in *Escherichia coli*. Subsequently, the synthesized *tauRD* DNA fragment was inserted into the plasmid pTYB12 (New England Biolabs) at the EcoRI-XhoI restriction sites, ensuring precise positioning for expression (Fig. 1b and Table 1). The DNA sequences of the constructs were validated using the dideoxy-chain termination method (Macrogen; http://dna.macrogen.com/kor/). The resulting expression plasmids were introduced into competent *E. coli* ER2566 cells (New England Biolabs) for subsequent protein expression. The protein was purified from isopropyl-β-D-thiogalactoside (IPTG) (Cat#: I1401-0025, Duchefa Biochemie)-induced *E. coli* ER2566 cultures (0.4 mM IPTG concentration) using affinity chromatography with a chitin bead column (New England Biolabs) following the manufacturer’s protocols. When higher purity was necessary for biochemical experiments, ion-exchange chromatography was employed to further increase the purity of the TauRD protein extracted from the chitin column after dialysis. The purity of the recombinant proteins was validated through sodium dodecyl sulfate-polyacrylamide gel electrophoresis (SDS-PAGE) and Western blot analysis using an anti-tau antibody (A-10; Santa Cruz Biotechnology) *(82)*. To produce a recombinant FlaB-TauRD fusion protein, we designed a pET30a+ plasmid (Novagen) containing a DNA fragment encoding TauRD (amino acids 243-368) (Table 1). The inserted DNA fragment was generated through PCR amplification utilizing a codon-optimized DNA template and the following primer pair: tauRD-F and tauRD-R, with EcoRI and XhoI sites, respectively (Table 2). For the construction of the recombinant FlaB-TauRD fusion, an additional primer set consisting of flaB-F and flab-R, was designed to amplify the FlaB DNA fragments (Table 2). The primers used were custom-synthesized with overhangs that are recognized by specific restriction enzymes (REs), namely, NdeI and EcoRI. The FlaB amplicons were subsequently digested using the appropriate REs and ligated to the N-terminus of TauRD, resulting in the fusion construct (FlaB-TauRD) within the pET30a+ vector. The accuracy of the DNA sequences within the expression vector was verified through dideoxy-chain termination sequencing (Macrogen). Subsequently, the resultant plasmid was introduced into competent *E. coli* BL21 cells. Protein expression was induced with 1 mM IPTG for 18 h at 20 °C, after which the cells were collected by centrifugation and stored at −80 °C until further use. The bacterial pellets were subsequently lysed in 50 ml of lysis buffer (pH 8) composed of 50 mM NaH_2_PO_4_, 300 mM NaCl, 10 mM imidazole, 0.1% Triton X-100, 0.1% Tween, and 20 µM phenylmethylsulfonyl fluoride. After centrifugation at 35,000 × *g* for 30 min, the resulting cell-free supernatant was loaded onto a column containing Ni-NTA agarose beads (Cat# 30210, Qiagen). The purity of the recombinant FlaB-TauRD protein was ascertained by SDS-PAGE and Western blot analysis, employing anti-FlaB or anti-TauRD serum raised in BALB/c mice. The purified proteins were resuspended in sterile phosphate-buffered saline (PBS) through extensive dialysis against PBS. To eliminate lipopolysaccharide (LPS) contamination, Triton^TM^ X-114 (Sigma-Aldrich) treatment was used, as described previously *(83)*. Residual traces of Triton X-114 were subsequently removed by incubation with Bio-Beads™ SM-2 (Bio-Rad Laboratories) at a ratio of 0.3 g of Bio-BeadsTM-2 per 1 ml of protein. The remaining LPS content was quantified using the gel-clotting agent Endosafe LAL Kit Pyrotell® (Cat# G5006-5, Cape Cod). The LPS concentrations in the protein preparations were maintained below the Food and Drug Administration (FDA) guidelines of 0.15 EU/30 g per mouse.

**Table 1:** Bacterial strains and plasmids used in this study

Bacteria	Description	Source
*Escherichia coli* DH5α	F^−^ *recA1* restriction negative	Laboratory collection
*E. coli* ER2566	F^-^ λ^-^ *fhuA2 [lon] ompT lacZ::T7 gene1 gal sulA11∆(mcrC-mrr)114::IS10R(mcr-73::miniTn10-TetS)2* *R(zgb210::Tn10)(*TetS*) endA1 [dcm]*	New England Biolabs, Inc.
*E. coli* BL 21 (DE3)	*hsdS gal (λcIts857 ind1 Sam7 nin5 lacUV5-T7 gene1)*	Laboratory collection
Plasmid		
pTYB12	N-terminal fusion expression vector in which the N terminus of a target protein is a fused Intein-tag; Ap^r^	New England Biolabs, Inc.
pET30a(+)	N-terminal fusion expression vector in which the N terminus of a target protein is a fused His-tag; Km^r^	EMD Bioscience
pTYB12::*flaB*	1.5-kb EcoRI-PstI fragment containing ORF of *flaB* cloned into pTYB12	^54^
pTYB12::*tauRD*	378-bp EcoRI-XhoI fragment containing ORF of *tauRD* encoding the 4R-tau repeat region (amino acid 243-368) from 2N4R-human tau cloned into pTYB12	This study
pET30a(+)::*flaB-tauRD*	pET-30a(+) plasmid containing a DNA-fragment of *flaB* fused with *tauRD* (NdeI-EcoRI-XhoI)	This study

**Table 2:** PCR primers used in this study

Primer	Nucleotide sequence (5′ to 3′)
tauRD-F	CCGGAATTCCTGCAAACAGCCCCGGTTCC
tauRD-R	CCGCTCGAGATTACCTCCTCCCGGCACAT
flaB-F	GGAATTCCATATGGCAGTGAATGTAAATAC
flaB-R	CCG GAATTCGCCTAGTAGACTTAGCGCTG

The recognition sites of the restriction enzyme are indicated by underlined sequences.

### Mice

Specific pathogen-free female BALB/c mice were purchased from Orient Bio, Inc. (Seongnam, Korea). BALB/c TLR5^−/−^ mice were previously characterized and described^77^. P301S-transgenic mice [B6; C3-Tg (Prnp-MAPT*P301S) PS19Vle/J]^78^ were obtained from the Jackson Laboratory.

### Ethics statement

All animal experimental procedures were performed with the approval of the Chonnam National University Institutional Animal Care and Use Committee under protocol CNU IACUC-H-2022-45. The maintenance of the animal research facility and experimental procedures strictly adhered to the guidelines of the Animal Welfare Act enacted by the Korean Ministry of Agriculture, Food and Rural Affairs.

### Intranasal immunization

To optimize the immunization schedule, 7-week-old female BALB/c mice (OrientBio, Seongnam, Korea) were intranasally immunized as follows: PBS (control group), PBS containing 10 μg of TauRD (TauRD), and PBS containing 10 μg of TauRD plus 4 μg of FlaB (TauRD+FlaB). The final volume administered for each vaccination was 20 μl per mouse. To compare the TauRD-specific antibody responses between TauRD+FlaB and the FlaB-TauRD fusion protein, BALB/c mice were anesthetized by intraperitoneal injection of Zoletil® 50 (Virbac corporation, Carros, France) and Rompun^TM^ (Elanco Animal Health Korea Co., Ltd.) and were intranasally immunized as follows: PBS (control group), PBS containing 3.9 μg of TauRD (TauRD), PBS containing 3.9 μg of TauRD plus 12 μg of FlaB (TauRD+FlaB), and 14 μg of the FlaB-TauRD fusion protein. This immunization regimen was repeated five times at 1-week intervals. For the active immunization of P301S mice with the FlaB-TauRD fusion protein, both male and female P301S mice aged 3 or 6 months were subjected to intranasal immunization. These mice were administered either PBS or 14 µg of FlaB-TauRD, for a total of 10 immunizations conducted at 1-week intervals. For the detection of TauRD-specific immune responses, blood samples were prepared from the euthanized mice by cervical dislocation.

### Determination of TauRD-specific antibody titers by ELISA

Ninety-six-well ELISA plates (Corning) were coated with TauRD at concentration of 2 µg/ml in PBS. Subsequently, serum samples from individual mice were added to separate wells, with 2-fold serial dilutions performed in blocking buffer. After incubation and thorough washing steps, anti-mouse IgG-specific horseradish peroxidase (HRP)-conjugated secondary antibodies (Cat# P0260, Dako) were added to the wells at 1:2000 dilution. To initiate the reaction, the HRP-specific substrate BD OptEIA™ (BD Biosciences) was applied, and the reaction was terminated using 2 N H_2_SO_4_. The absorbance was measured at 450 nm using a microplate reader (SpectraMAX190, Molecular Devices).

### Characterization of TauRD by Western blot analysis

TauRD was loaded onto a 12% SDS-PAGE gel after boiling for 5 min and subsequently transferred onto nitrocellulose membranes. For native PAGE and subsequent Western blot analysis, TauRD was mixed with native sample buffer (Bio-Rad Laboratories) and loaded onto a 10% PAGE gel. Before membrane transfer, the PAGE gel was immersed in 50 mM Tris-HCl buffer (pH 7.5) containing 1% SDS for 10 min. The primary antibodies used for Western blotting included anti-Tau A-10 (1:500, sc-390476; Santa Cruz Biotechnology), anti-TauRD+FlaB serum obtained by intranasal immunization with 10 μg of TauRD plus 4 μg of FlaB, and anti-Tau_sp_mix+FlaB serum obtained by intranasal immunization of 100 μg of Tau_sp_mix (Tau_sp_1, Tau_sp_2, Tau_sp_3, and Tau_sp_4; 25 μg each) combined with 4 μg of FlaB. Uncropped and unprocessed scans of the SDS-PAGE, native PAGE, and blots are provided in the Source Data file.

### Immunofluorescence analysis of TauRD aggregates

The purified TauRD protein was fibrillized by incubation at 50 µM in PBS (pH 7.4) in the presence of 2 mM dithiothreitol (DTT) (Cat# 0281-5 G; VWR Life Science) and the anionic cofactor heparin (heparin sodium salt from porcine intestinal mucosa; Sigma, Cat# H3149) at a ratio of 1:8 (heparin:TauRD) for 5 days at 37 °C, without agitation^79^. At various time points during the incubation, protein samples were deposited onto glass coverslips that had been precoated with poly-D-lysine. Following fixation with 4% paraformaldehyde, the protein samples were stained with 0.01% thioflavin S (Sigma) and subsequently subjected to immunofluorescence staining. Immunofluorescence staining was carried out using TauRD antiserum obtained from mice immunized with FlaB+TauRD, and an Alexa Fluor® 546-conjugated secondary antibody derived from goat anti-mouse IgG (1:200, Cat# A11003; Invitrogen).

### Transmission electron microscopy (TEM)

Fibrillization of the TauRD protein was initiated at 37 °C in the presence of the anionic cofactor heparin, following the procedure described above. Aggregation was allowed to proceed for 3 or 5 days. Subsequently, solutions of the TauRD protein were diluted 0.1 mg/ml and applied to carbon-coated copper grids. These grids were then stained with 2% uranyl acetate. The prepared samples were examined using a transmission electron microscope (JEM−1400; JEOL Ltd., Japan) for detailed structural analysis.

### TauRD aggregate uptake assays by BV2 microglial cell line

BV2 cells were generously provided by Dr. Changjong Moon of Chonnam National University (Gwangju, Korea). Fibrillization of the TauRD protein was induced in the presence of heparin for 3 days. Subsequently, 50 µM TauRD fibrils were sonicated for 10 s for fragmentation and were then incubated with the antiserum for 2 h at room temperature with gentle agitation. BV2 cells were cultured in DMEM (Gibco) supplemented with 10% fetal bovine serum (FBS; Cat# 16000044; Gibco) and plated at a density of 10^5^ cells per well in a 24-well plate. On the following day, the cell culture medium was replaced with fresh DMEM containing 0.5% FBS, and the above TauRD-antibody complexes were added to the BV2 cells.

For microscopic imaging, TauRD fibrils were conjugated with Alexa Fluor^TM^ 488 (1:200, Cat# A32723, Invitrogen) before sonication. These conjugated fibrils were then incubated with the antiserum as described above. TauRD-antibody complexes were applied to BV2 cells for 30 min. Following internalization, the cells trypsinized, replated onto glass coverslips, and fixed using 4% paraformaldehyde. The BV2 cells were subsequently stained with wheat germ agglutinin (WGA)-Alexa Fluor^TM^ 555 conjugate (1:200, Cat# 11590816, Invitrogen) and 4′,6-diamidino-2-phenylindole (DAPI, Thermo Scientific) for visualization.

### Primary mouse microglial culture and TauRD aggregate uptake assays

The cortices from P0-P3 C57BL/6 mouse pups were dissected and the meninges were stripped under a microscope. The cortices were mechanically dissociated by gentle pipetting. After centrifugation at 300 × *g* for 5 min, the cells were seeded into poly-D-lysine (PDL)-coated 75-cm^2^ cell culture flasks. Mixed glia was cultured in DMEM containing 10% FBS and 1% penicillin-streptomycin at 37 °C in a 5% CO_2_ incubator, with one-third of the culture medium replaced with fresh medium twice a week. The microglia were isolated from the astrocyte layer 13–16 days later by mild trypsinization^80^. The medium was centrifuged for cell pelleting. Cells were seeded onto PDL-coated coverslips in 24-well plates (1 × 10^5^ cells/well) for immunofluorescence staining. Coverslips were washed with PBS and then fixed in 4% formaldehyde and permeabilized with 0.3% Triton X-100 in 1% BSA (bovine serum albumin). After blocking with 10% normal donkey serum in PBS, the cells were incubated with rabbit anti-Iba1 antibody (1:500, Wako, 019-19741) diluted with 1% BSA in PBS overnight at 4 °C. After rinsing three times with PBS for 5 min, the cells were incubated with Alexa Fluor^TM^ 594-conjugated donkey anti-rabbit secondary antibodies (1:500, Invitrogen, A-21207) for 1 h at room temperature. Nuclei were counterstained with DAPI and further stained with 0.01% thioflavin S. Coverslips were then mounted with the ProLong^TM^ Gold Antifade Mountant with DAPI. The uptake of TauRD per cell was quantified by determining the percentage of the ThS area overlapped with Iba-1 positive cells using Image J (National Institutes of Health, Bethesda, MD, USA).

### Immunohistochemical analysis of brain tissue

Mice were deeply anesthetized and transcardially perfused with 15 ml of PBS. The brains were removed and fixed for 24 h with 10% formalin, washed with PBS, embedded in paraffin, and cut into 6-µm thick coronal sections. The human brain tissue samples used in this study were obtained at autopsy, fixed in ethanol or paraformaldehyde, embedded in paraffin, and cut into 6 μm thick sections. For IHC staining, the brain sections were deparaffinized in xylene two times (5 min each) and rehydrated in 100% ethanol two times (3 min each) 95%, 80%, and 70% ethanol (3 min each). Endogenous peroxidase activity was quenched by incubating the slides in 3% H_2_O_2_ in methanol for 30 min, after which the slides were rinsed with PBS. Next, antigen retrieval was performed by boiling the slides in pH 6.0 citrate buffer. To reduce the nonspecific binding of primary antibodies to tissue, the slides were blocked with 10% BSA for 30 min. A primary antibody against pSer-202/pThr-205-tau (AT8, 1: 500, MN1020, Thermo Fisher Scientific), anti-TauRD+FlaB, or anti-FlaB+Tau_sp_mix was used in 5% bovine serum albumin (BSA) in PBS buffer and incubated at 4 °C overnight inside a wet chamber. After washing with 0.05% Tween 20 in PBS, the sections were sequentially incubated with an HRP-conjugated anti-mouse secondary antibody (1:1000, Cat# P0260, Dako) for 1 h. The bound antibody complexes were visualized by incubating the slides in DAB substrate (Cat# K3468, Dako) and counterstaining them with hematoxylin (Abcam). The slides were scanned with a 20× objective using a slide scanner (Axioscan7, Zeiss).

### Disease scoring of P301S mice

To assess phenotypic changes in P301S mice following vaccination, disease scores were assigned based on the scoring system presented in Table 3. Scoring was conducted in a blinded fashion by two independent researchers utilizing the stated criteria to ensure objectivity and consistency in the assessment process.

**Table 3:** Arbitrary disease scoring of P301S transgenic mice

Score	Characteristics
0	Normal
2	Hind leg clasping
4	Hunched back
6	Hunched back with reduced activity
8	Weak hind limb and impaired forward movement
10	Paralysis of hind limbs with impaired forward movement
11	Total paralysis
12	Death

### Live imaging showing the inhibition of fibrillization by antibodies raised in P301S transgenic mice

To test the anti-fibrillization effect of the antibodies, IgG antibodies were purified from serum samples using an IgG Purification Kit (Cat# 45206; Thermo Fisher Scientific) following the manufacturer’s instructions. Fifty micrograms of purified TauRD protein in 300 μl of PBS was incubated with purified IgG antibodies at a final concentration of 0.04 mg/ml at 4 °C overnight with end-over-end rotation. After overnight incubation, the anionic cofactor heparin (Cat# H3149-25KU; Sigma-Aldrich) was added at an 8:1 ratio as described above to promote TauRD aggregation, and real-time live imaging was carried out using an EVOS FL Auto 2 system (Invitrogen, AMAFD2000). After setting the magnification, focus, and channel illumination parameters, an automated image acquisition routine was used to scan the 96 well plates by autofocusing on protein aggregates. Nine fields per well were scanned and imaged every 3 h for 96 h. A total of 288 images were obtained and further used in video generation.

### Mouse behavioral test

For mouse behavioral tests, 6-month-old P301S mice were intranasally immunized ten times at one-week intervals with PBS [male (*n* = 3) and female (*n* = 5)] or 14 μg of the FlaB-TauRD fusion protein [male (*n* = 3) and female (*n* = 5)]. Fourteen weeks after the last round of immunization, an open field test, a novel objective recognition test (NORT), and a Y-maze test were performed. For the open-field test, mice were allowed to move in an open-field arena (36 × 36 cm, 40 cm high) made of hard plastic board^81^. Locomotor activity was assessed using a camera placed above the arena and an automated video tracking system (ANY-MAZE software; Stoelting). Locomotor activity was analyzed as the total distance traveled for the first 20 min. NORT was performed as previously described^81^. Mice were introduced to two similar objects (A) located in the arena during training for 8 min^81^. After 24 h, the mice were placed in the arena with the same object and a novel object (B). The sniffing time near each object was measured using a camera and ANY-MAZE software for 8 min. The preference index (%) was calculated as follows: [new object (B) time/both objects (A + B) time] × 100. The Y-maze test was performed as previously described^82^. Briefly, mice were placed in a Y-maze with three arms (length: 40 cm, width: 5 cm, height: 13 cm) and allowed to freely explore the maze for 7 min. The number of entries to each arm was analyzed with a video tracking system (ANY-MAZE software). The alternation ratio was calculated as follows: [the number of spontaneous alternations/total arm entries − 2] × 100. Mice with more than five arm entries were included in the analysis.

### Western blot analysis of tau protein in the P301S mouse brain

The right hemisphere of the brain was dissected to isolate proteins. Brain tissues were lysed in 5 ml of RIPA buffer (Cat#89900, Thermo Scientific) containing 2 mM proteinase inhibitor (Cat#78830, Sigma Aldrich). The lysate was sonicated for 2 min on ice and then centrifuged at 1100 × *g* for 15 min. Following this initial centrifugation, the supernatants were subjected to a second centrifugation at 21,000 × *g* for 30 min. The protein concentration in the resulting supernatants was determined using a BCA Kit (Cat#23225, Thermo Scientific). Subsequently, Western blotting was performed following the previously described protocol^83,84^. The primary antibody employed for Western blotting was anti-Tau A-10 (1:500, sc-390476; Santa Cruz Biotechnology).

### Statistical analysis

Statistical analyses were conducted using Prism 8.0.2 software for Windows (GraphPad Software). The results are presented as the means ± standard errors of the means (SEMs) unless otherwise specified. Comparisons between two groups were performed using either Student’s *t* test or analysis of variance (ANOVA). TauRD uptake by primary glial cells was analyzed by two-way ANOVA analysis followed by Tukey’s multiple comparisons test using GraphPad Prism version 10 for Mac (GraphPad, La Jolla, CA). Survival differences were assessed for statistical significance using the Gehan-Breslow-Wilcoxon test. A *P* value of less than 0.05 was considered statistically significant.

### Reporting summary

Further information on research design is available in the Nature Research Reporting Summary linked to this article.

### Supplementary information

Supplementary Information

reporting summary

Supplementary Video 1a

Supplementary Video 1b

## Data Availability

All data are available in the main text or the supplementary materials.
