# Author Correction: Development of an anti-tauopathy mucosal vaccine specifically targeting pathologic conformers

**DOI:** 10.1038/s41541-024-00910-3

**Published:** 2024-06-28

**Authors:** Wenzhi Tan, Jayalakshmi Thiruppathi, Seol Hee Hong, Sao Puth, Sophea Pheng, Bo-Ram Mun, Won-Seok Choi, Kyung-Hwa Lee, Hyun-Sun Park, Duc Tien Nguyen, Min-Cheol Lee, Kwangjoon Jeong, Jin Hai Zheng, Young Kim, Shee Eun Lee, Joon Haeng Rhee

**Affiliations:** 1 Clinical Vaccine R&D Center, Chonnam National University, Hwasun-gun, Republic of Korea; 2 Department of Microbiology, Chonnam National University Medical School, Hwasun-gun, Republic of Korea; 3 Combinatorial Tumor Immunotherapy MRC, Chonnam National University Medical School, Hwasun-gun, Republic of Korea; 4 National Immunotherapy Innovation Center, Hwasun-gun, Republic of Korea; 5 Department of Pharmacology and Dental Therapeutics, School of Dentistry, Chonnam National University, Gwangju, Republic of Korea; 6 School of Biological Sciences and Technology, Chonnam National University, Gwangju, Republic of Korea; 7 Department of Pathology, Chonnam National University Medical School, Hwasun-gun, Republic of Korea; 8 Department of Pharmacology, Chonnam National University Medical School, Hwasun-gun, Republic of Korea; 9 Seegene Inc, Seoul, Republic of Korea; 10 Department of Oral Pathology, School of Dentistry, Chonnam National University, Gwangju, Republic of Korea; 11 Present Address: School of Biomedical Sciences, Hunan University, Changsha, China

**Keywords:** Adjuvants, Alzheimer's disease

Correction to: *npj Vaccines* (2024) **9**:108; 10.1038/s41541-024-00904-1, published online 15 June 2024

“In this article the grant number RS-2022-KH127978(HV22C0079 relating to Korea Health Technology R&D Project through the Korea Health Industry Development was omitted. The original article has been corrected.”

