# Supplementary material for: Development of an anti-tauopathy mucosal vaccine specifically targeting pathologic conformers
Source: NPJ Vaccines. 2024 Jun 15;9:108. doi: 10.1038/s41541-024-00904-1 (PMC11180213; doi:10.1038/s41541-024-00904-1)

Supplementary Materials

Materials and Methods

Peptides

The peptide immunogens tau253-262 ($Tau_{sp}1$), tau284-293 ($Tau_{sp}2$), tau319-328 ($Tau_{sp}3$), and tau347-356 ($Tau_{sp}4$) were synthesized by the AnyGen Company (Gwangju, Korea) with a purity of >97% (Supplementary Table 1).

Intranasal immunization with the Tau peptide mixture

Five mice per group were vaccinated with various combinations of antigen and adjuvant. For TauRD dose optimization, 6-week-old female BALB/c mice (OrientBio, Seongnam, Korea) were immunized with phosphate-buffered saline (PBS, negative control group), 100 µg of T_{sp} mixture ($Tau_{sp}1$, $Tau_{sp}2$, $Tau_{sp}3$, or $Tau_{sp}4$; 25 µg each), or 100 µg of T_{sp} mixture and with 4 µg of FlaB, at one-week intervals. The final volume for each dose was 20 µl per mouse. For active immunization of P301S mice with the FlaB-TauRD fusion protein, both male and female P301S mice (ten to eleven mice per group) were intranasally immunized with PBS or 14 µg of FlaB-TauRD 10 times at one-week intervals beginning 3 or 6 months after birth.

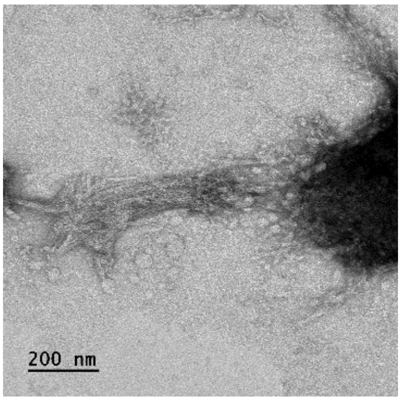

Supplementary Figure 1. Transmission electron micrographs of TauRD proteins. Transmission electron micrographs depict the structural characteristics of TauRD protein samples. TauRD protein was incubated at 37 ℃ for 5 days before being subjected to transmission electron microscopy (TEM) analysis.

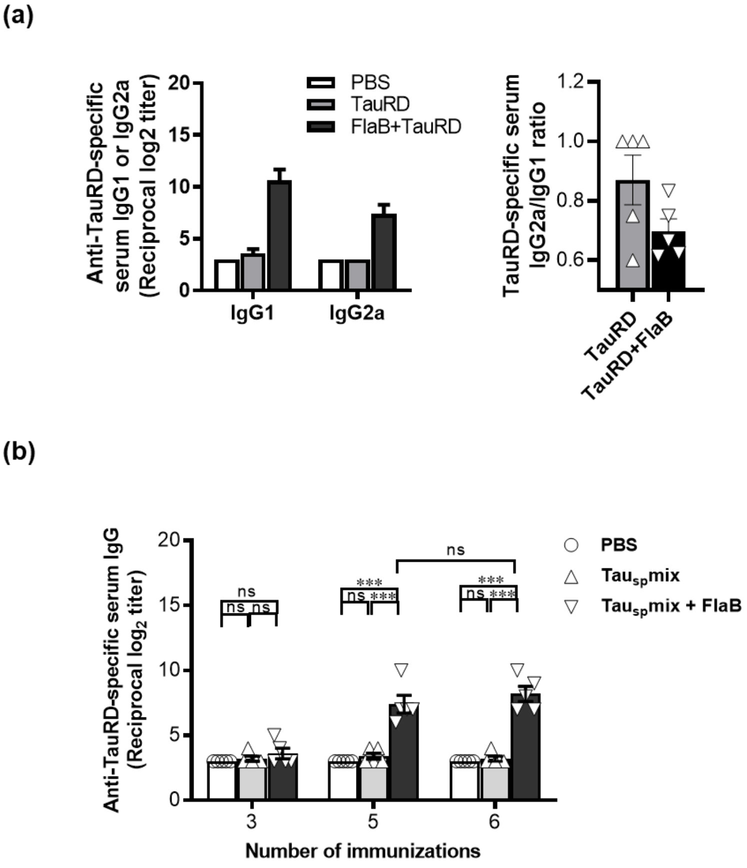

Supplementary Figure 2. TauRD-specific antibody response following intranasal immunization with FlaB-adjuvanted Tau$_{sp}$ mixture. (a) Determination of TauRD-specific IgG$_{2a}$/IgG$_1$ ratio. Wild-type BALB/c mice were subjected to intranasal administration at one-week intervals: PBS, PBS containing 10 µg of TauRD (TauRD), PBS containing 10 µg of TauRD and 4 µg of FlaB (TauRD+FlaB). Following the fifth immunization, the levels of TauRD-specific serum IgG1 and IgG2a were evaluated using enzyme-linked immunosorbent assay (ELISA). **(b)** Determination of TauRD-specific antibody titer measured by ELISA. Wild-type BALB/c mice were subjected to intranasal administration of one of the following at one-week intervals: PBS, PBS containing 100 µg of Tau$_{sp}$ mixture (Tau$_{sp}$1, Tau$_{sp}$2, Tau$_{sp}$3, Tau$_{sp}$4; 25 µg each), or PBS containing 100 µg of Tau$_{sp}$ mixture and 4 µg of FlaB (TauRD+FlaB). Following the third, fifth, and sixth immunizations, the levels of TauRD-specific serum IgG were evaluated using enzyme-linked immunosorbent assay (ELISA).

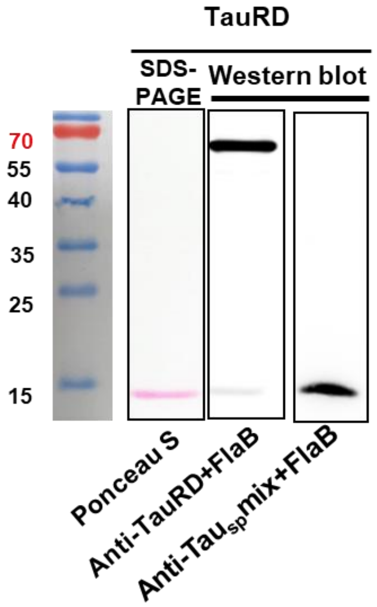

Supplementary Figure 3. Immunoblot analysis of recombinant TauRD protein using anti-TauRD+FlaB serum induced by intranasal immunization. Recombinant TauRD proteins were resolved using SDS-PAGE, and subsequently, the TauRD protein bands were detected and visualized using antiserum induced by intranasal immunization with TauRD and FlaB (1:500 dilution**).** Postimmune serum obtained from the FlaB+Tau$_{sp}$mix group at a 1:200 dilution was employed as an antibody capable of recognizing the sequence-specific Tau protein.

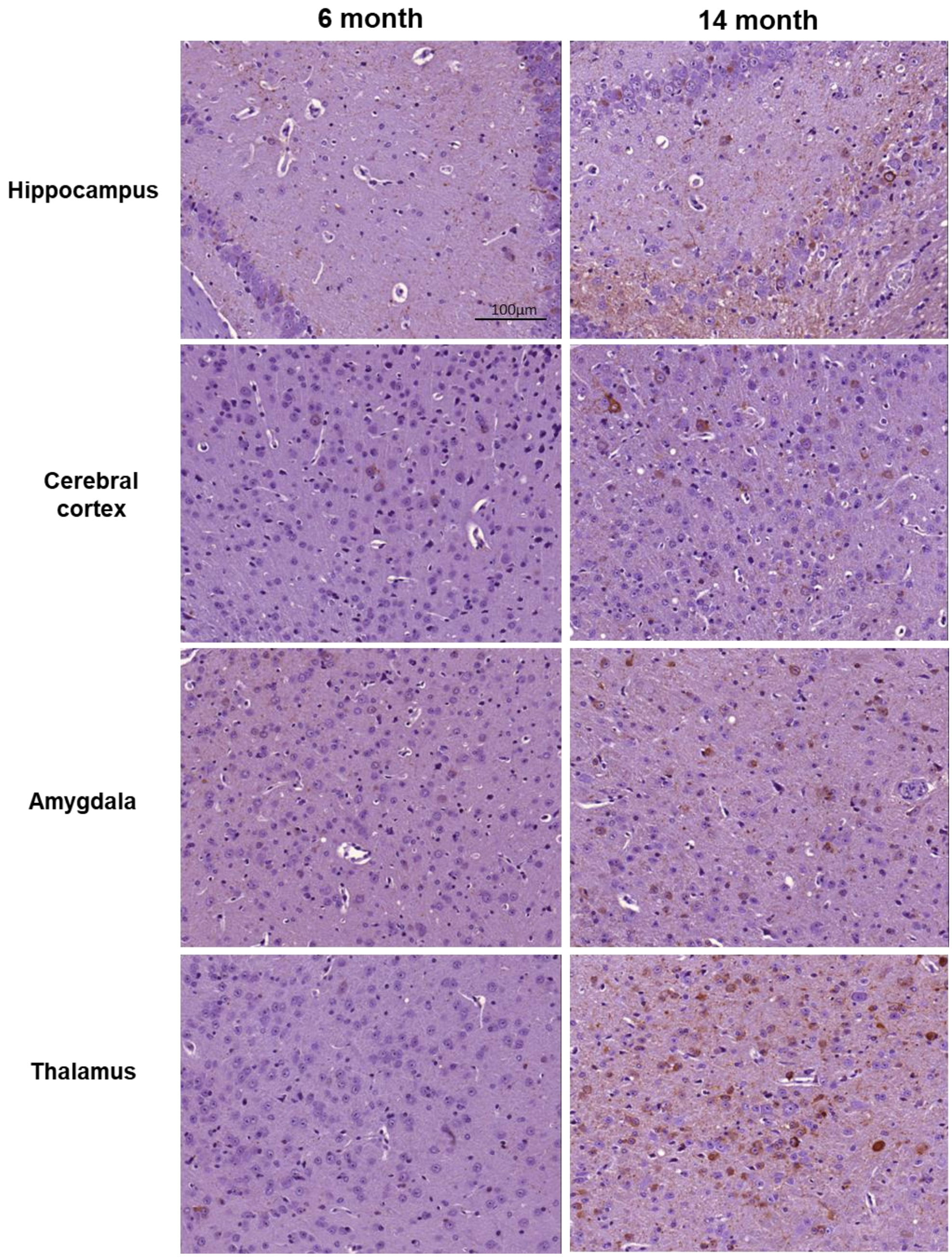

Supplementary Figure 4. Immunohistochemical analysis of hyperphosphorylated tau using AT8 antibody in P301S transgenic mice. Immunohistochemical analysis of hyperphosphorylated tau using AT8 antibody in the hippocampus, cerebral cortex, amygdala, and thalamus sections of 6- and 14-month-old P301S transgenic mice.

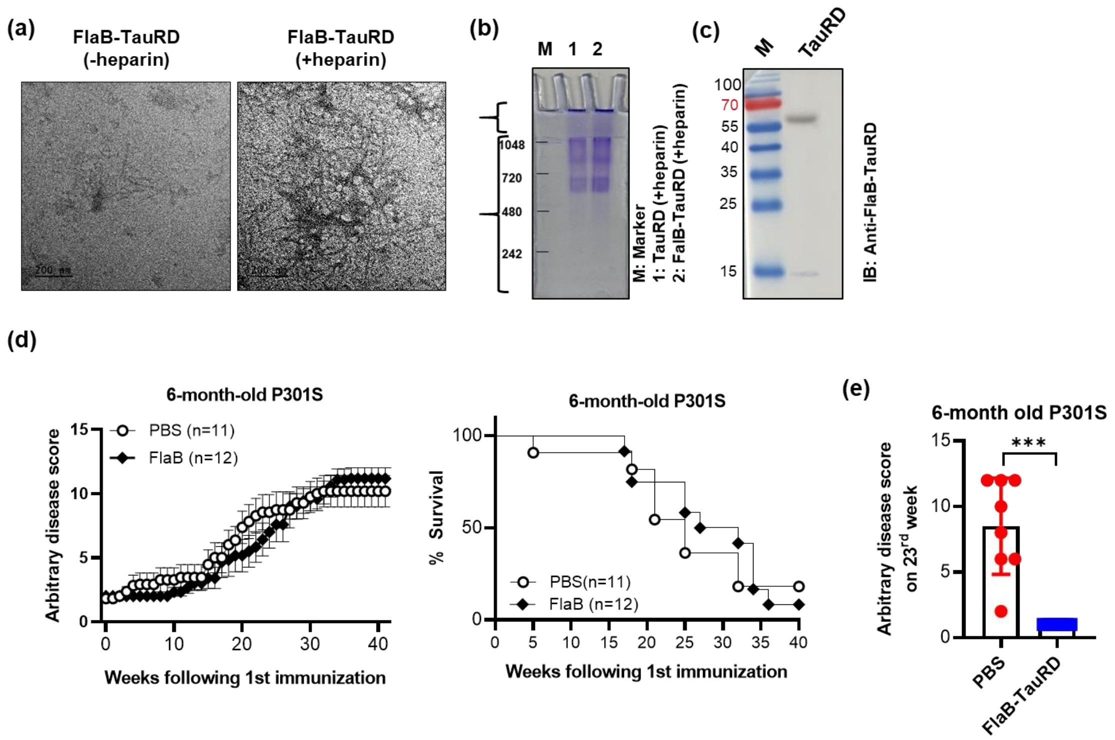

Supplementary Figure 5. Biochemical and functional characteristics of built-in adjuvanted FlaB-TauRD fusion protein vaccine. (a) Transmission electron micrographs of FlaB-TauRD protein. Transmission electron micrographs of TauRD proteins aggregated via heparinization. The fibrillization of TauRD was initiated at 37 °C in the presence or absence of the anionic cofactor heparin at a TauRD:heparin molar ratio of 8:1. The aggregation was allowed to proceed for 3 days. **(b)** Analysis of purified recombinant FlaB-TauRD by native-PAGE. Recombinant FlaB-TauRD was resolved using native-PAGE. **(c)** characterization of anti-FlaB-tauRD serum. Recombinant TauRD proteins were resolved using SDS-PAGE, and subsequently, the TauRD protein bands were detected and visualized using antiserum induced by intranasal immunization with FlaB-TauRD. **(d)** Measurement of the disease score and survival of vaccinated mice. Following ten rounds of immunization with PBS or FlaB, the disease score and survival of the immunized P301S mice were assessed. **(e)** TauRD-specific serum IgG levels in P301S mice. Six-month-old P301S mice were intranasally immunized with phosphate-buffered saline (PBS) and 14 µg of FlaB-TauRD 10 times at intervals of 1 week. Fourteen weeks after the tenth round of immunization, the levels of TauRD-specific serum IgG were evaluated using an enzyme-linked immunosorbent assay (ELISA).

Supplementary Table 1. Amino acid sequences of Tau short peptide (Tau_{sp})

Selected short peptides	Amino acid sequence
Tau_{sp}1 (253-262)	L K N V K S **K I G S**
Tau_{sp}2 (284-293)	L S N V Q S **K C G S**
Tau_{sp}3 (319-328)	T S **K C G S** L G N I
Tau_{sp}4 (347-356)	K D R V Q S **K I G S**

Supplementary Movie 1. Live imaging of TauRD aggregation. An assay of aggregation inhibition was conducted using IgG purified from pooled anti-sera obtained after the 10th immunization. TauRD protein was pre-treated with IgG purified from pooled anti-PBS **(A)** or anti-FlaB-TauRD **(B)** serum obtained from P301S transgenic mice after the 10 rounds of immunization. Subsequently, TauRD aggregation was induced in the presence of heparin over a 3-day period. TauRD protein aggregation was analyzed utilizing the EVOS FL Auto 2 imaging system. TauRD aggregation was quantified by acquiring images automatically at 3-hour intervals for a total duration of 96 hours. At each time point, 9 fields per sample were imaged, resulting in the acquisition of a total of 288 images, which were then compiled into a video for subsequent analysis. The anti-FlaB-TauRD serum effectively inhibited TauRD aggregate formation while quaternary structure of TauRD aggregate were observed in anti-PBS treated wells.

Figure 1

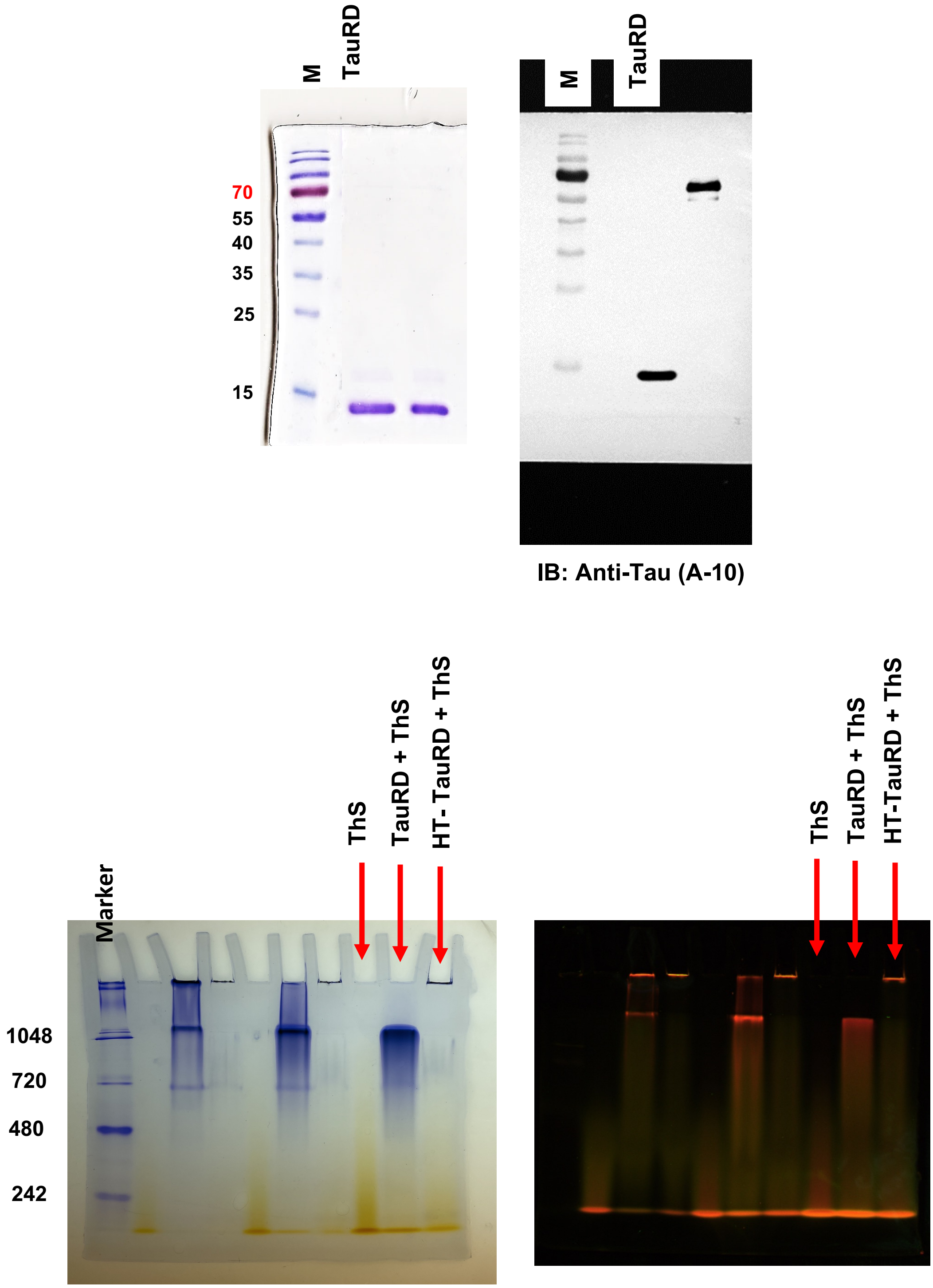

Coomassie brilliant blue

FOBI image (NeoScience)

* HT-TauRD: heparin-treated TauRD

Figure 3

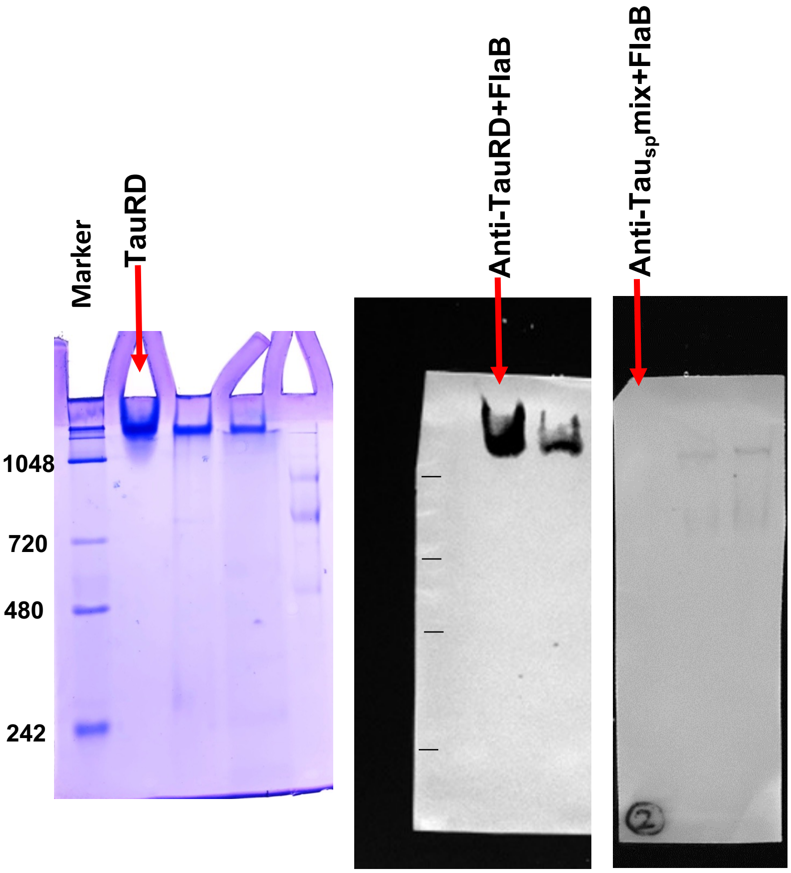

Heparin-treated TauRD
(Native PAGE)

Western blot

Heparin-treated TauRD
(Native PAGE)

Figure 5

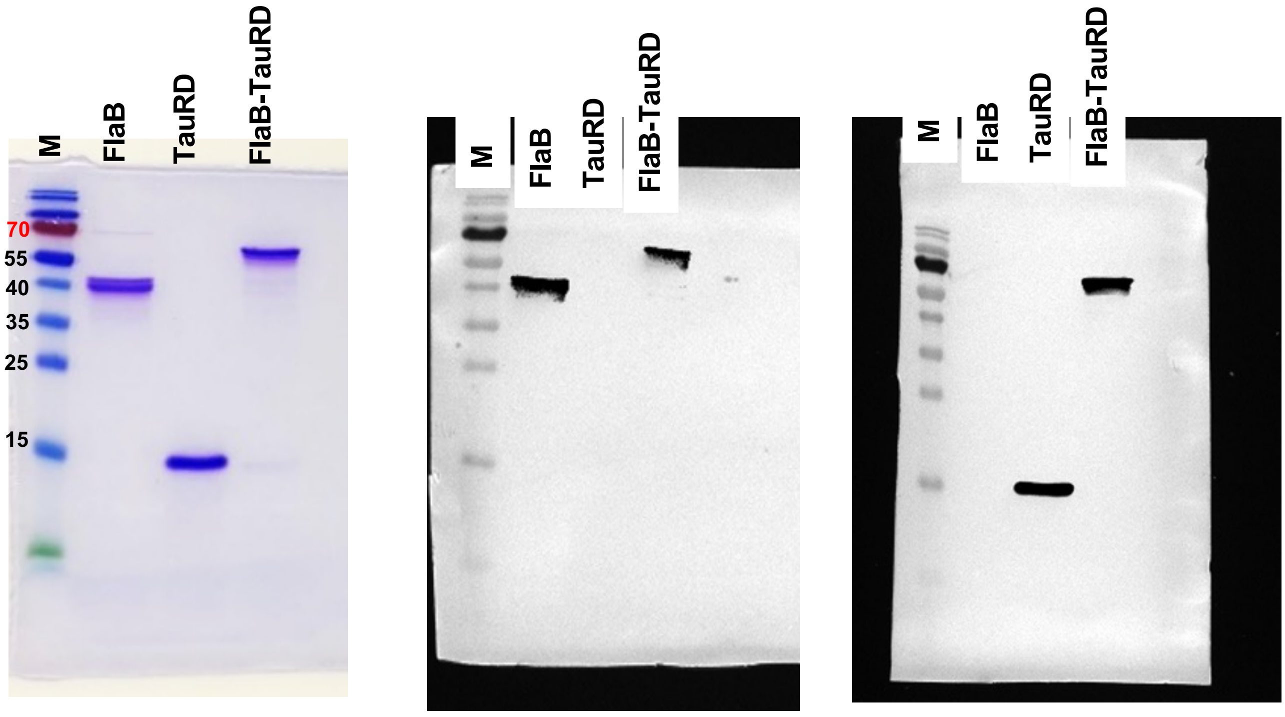

SDS-PAGE IB: Anti-FlaB IB: Anti-TauRD

Figure 7

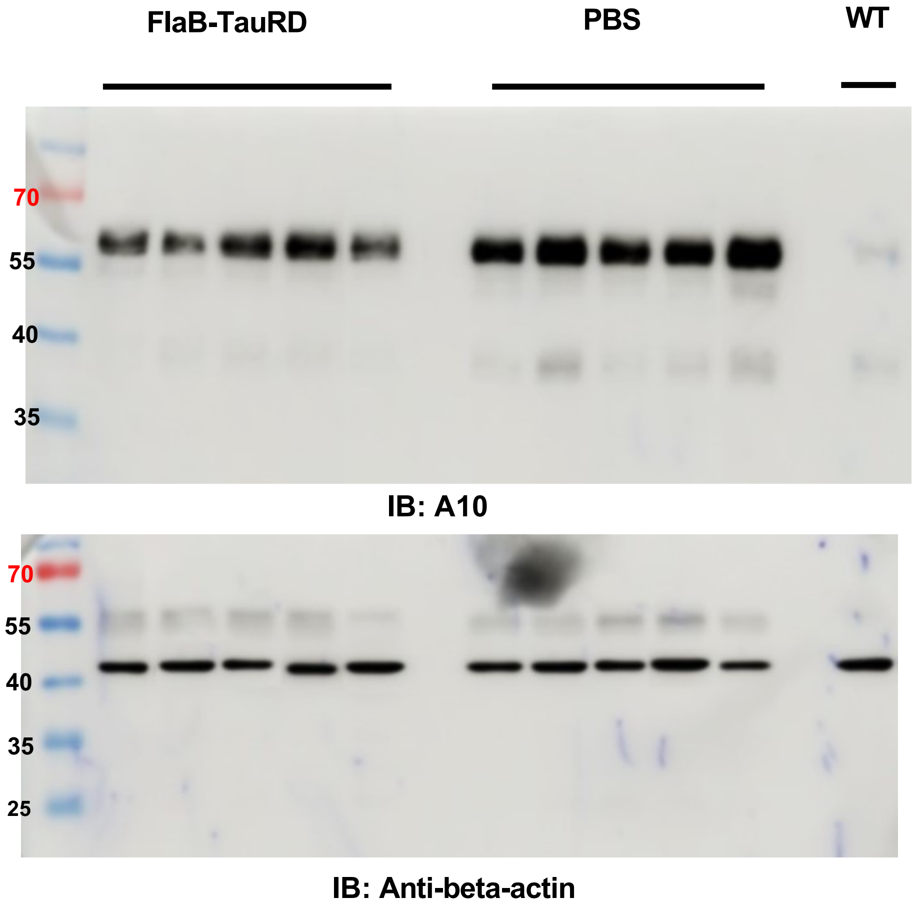
FlaB-TauRD
PBS
WT
70
55
40
35
IB: A10
70
55
40
35
25
IB: Anti-beta-actin

Supplementary Figure 5

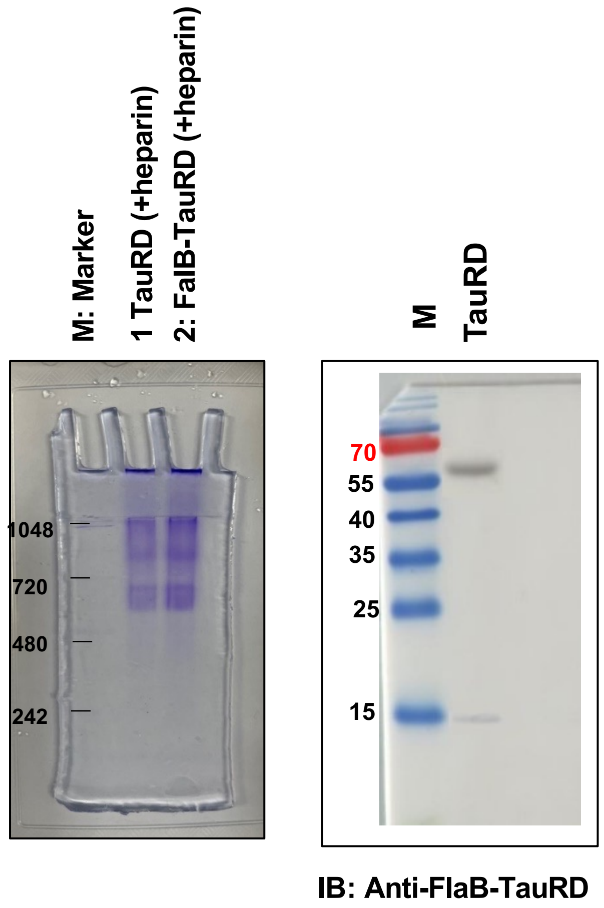

Supplement: Supplementary file 1 — Supplementary Information [file 41541_2024_904_MOESM1_ESM.pdf]
