# Supplementary material for: Development of an anti-tauopathy mucosal vaccine specifically targeting pathologic conformers
Source: NPJ Vaccines. 2024 Jun 15;9:108. doi: 10.1038/s41541-024-00904-1 (PMC11180213; doi:10.1038/s41541-024-00904-1)
Supplement: Supplementary file 2 — reporting summary [file 41541_2024_904_MOESM2_ESM.pdf]

nature portfolio

Corresponding author(s): Joon Haeng Rhee and Shee Eun Lee

Last updated by author(s): Feb 29, 2024

Reporting Summary

Nature Portfolio wishes to improve the reproducibility of the work that we publish. This form provides structure for consistency and transparency in reporting. For further information on Nature Portfolio policies, see our Editorial Policies and the Editorial Policy Checklist.

Statistics

For all statistical analyses, confirm that the following items are present in the figure legend, table legend, main text, or Methods section.

n/a	Confirmed	
☐	☒	The exact sample size (*n*) for each experimental group/condition, given as a discrete number and unit of measurement
☐	☒	A statement on whether measurements were taken from distinct samples or whether the same sample was measured repeatedly
☐	☒	The statistical test(s) used AND whether they are one- or two-sided<br>*Only common tests should be described solely by name; describe more complex techniques in the Methods section.*
☐	☒	A description of all covariates tested
☐	☒	A description of any assumptions or corrections, such as tests of normality and adjustment for multiple comparisons
☐	☒	A full description of the statistical parameters including central tendency (e.g. means) or other basic estimates (e.g. regression coefficient) AND variation (e.g. standard deviation) or associated estimates of uncertainty (e.g. confidence intervals)
☐	☒	For null hypothesis testing, the test statistic (e.g. *F*, *t*, *r*) with confidence intervals, effect sizes, degrees of freedom and *P* value noted<br>*Give P values as exact values whenever suitable.*
☒	☐	For Bayesian analysis, information on the choice of priors and Markov chain Monte Carlo settings
☒	☐	For hierarchical and complex designs, identification of the appropriate level for tests and full reporting of outcomes
☒	☐	Estimates of effect sizes (e.g. Cohen's *d*, Pearson's *r*), indicating how they were calculated

Our web collection on statistics for biologists contains articles on many of the points above.

Software and code

Policy information about availability of computer code

Data collection

1)Statistical analysis: Graphpad Prism 8.0.2 software for Windows Prism version 10 software for Mac
2)Western blot analysis: iBrightTM CL1000,Firmware version: 1.2.5
3)Elisa analysis: Spectrophotometer (SpectraMax 190, Molecular Devices Crop., Menlo Park, CA)
4)Protein aggregation imaging: Transmission electron microscope (JEM−1400; JEOL 641 Ltd., Japan)
5)Immunohistochemical analysis: a 20x objective using a slide scanner (Axioscan7, Zeiss)
6)Real time image: carried out using an EVOS FL Auto 2 system (Invitrogen, AMAFD2000)
7)Mice behavioral test: assessed using a camera placed above the arena and an automated video tracking system (ANY-MAZE software; Stoelting).

Data analysis

This paper, all statistical analysis were calculated by using Graphpad Prism 8.0.2. Except,TauRD uptake by primary glial cells were analyzed by two-way ANOVA analysis followed by Tukey's multiple comparisons test using GraphPad Prism version 10 (GraphPad, La Jolla, CA). iBrightTM CL1000,Firmware version: 1.2.5 was used for western blot analysis. The ELISA analysis was read by using Spectrophotometer (SpectraMax 190, Molecular Devices Crop., Menlo Park, CA). Protein aggregation images were captured by transmission electron microscope (JEM−1400; JEOL Ltd., Japan) for detailed structural analysis. Immunohistochemical analysis of mice brain tissue were scanned with a 20x objective using a slide scanner (Axioscan7, Zeiss). To show the live image of the inhibition of fibrillization by antibodies raised in P301S transgenic mice analyses was carried out using an EVOS FL Auto 2 system (Invitrogen, AMAFD2000). Mice behavioral test has been assessed using a camera placed above the arena and an automated video tracking system (ANY-MAZE software; Stoelting).

For manuscripts utilizing custom algorithms or software that are central to the research but not yet described in published literature, software must be made available to editors and reviewers. We strongly encourage code deposition in a community repository (e.g. GitHub). See the Nature Portfolio guidelines for submitting code & software for further information.

Data

Policy information about availability of data

All manuscripts must include a data availability statement. This statement should provide the following information, where applicable:

- Accession codes, unique identifiers, or web links for publicly available datasets
- A description of any restrictions on data availability
- For clinical datasets or third party data, please ensure that the statement adheres to our policy

Provide your data availability statement here.

Research involving human participants, their data, or biological material

Policy information about studies with human participants or human data. See also policy information about sex, gender (identity/presentation), and sexual orientation and race, ethnicity and racism.

Reporting on sex and gender	*Use the terms sex (biological attribute) and gender (shaped by social and cultural circumstances) carefully in order to avoid confusing both terms. Indicate if findings apply to only one sex or gender; describe whether sex and gender were considered in study design; whether sex and/or gender was determined based on self-reporting or assigned and methods used. Provide in the source data disaggregated sex and gender data, where this information has been collected, and if consent has been obtained for sharing of individual-level data; provide overall numbers in this Reporting Summary. Please state if this information has not been collected. Report sex- and gender-based analyses where performed, justify reasons for lack of sex- and gender-based analysis.*
Reporting on race, ethnicity, or other socially relevant groupings	*Please specify the socially constructed or socially relevant categorization variable(s) used in your manuscript and explain why they were used. Please note that such variables should not be used as proxies for other socially constructed/relevant variables (for example, race or ethnicity should not be used as a proxy for socioeconomic status). Provide clear definitions of the relevant terms used, how they were provided (by the participants/respondents, the researchers, or third parties), and the method(s) used to classify people into the different categories (e.g. self-report, census or administrative data, social media data, etc.) Please provide details about how you controlled for confounding variables in your analyses.*
Population characteristics	*Describe the covariate-relevant population characteristics of the human research participants (e.g. age, genotypic information, past and current diagnosis and treatment categories). If you filled out the behavioural & social sciences study design questions and have nothing to add here, write "See above."*
Recruitment	*Describe how participants were recruited. Outline any potential self-selection bias or other biases that may be present and how these are likely to impact results.*
Ethics oversight	*Identify the organization(s) that approved the study protocol.*

Note that full information on the approval of the study protocol must also be provided in the manuscript.

Field-specific reporting

Please select the one below that is the best fit for your research. If you are not sure, read the appropriate sections before making your selection.

☒ Life sciences ☐ Behavioural & social sciences ☐ Ecological, evolutionary & environmental sciences

For a reference copy of the document with all sections, see nature.com/documents/nr-reporting-summary-flat.pdf

Life sciences study design

All studies must disclose on these points even when the disclosure is negative.

Sample size	We performed preliminary experiments to determined sufficient sample size for each experiment set.
Data exclusions	No sample or animal were excluded from analysis.
Replication	all experiment finding were performed at least 2 times reproducibly. The number of samples for each experiment indicated in figure legend. The data shown in the figure panels are the mean of all independent repeated experiments. SDS-PAGE and western blot pictures are from a representative experiment. all repeated experiments were successful.
Randomization	For invitro experiment, cells culture were chosen for different treatment randomly and all experiments were performed at least 2 times. For animal experiments, mice were breaded from our own facility for Intranasal immunization.
Blinding	ELISA and Western Blot assay was performed by individuals (Seol Hee Hong and Jayalakshmi Thiruppathi) were blinded to nature of mice under analysis.

Reporting for specific materials, systems and methods

We require information from authors about some types of materials, experimental systems and methods used in many studies. Here, indicate whether each material, system or method listed is relevant to your study. If you are not sure if a list item applies to your research, read the appropriate section before selecting a response.

Materials & experimental systems

n/a	Involved in the study
☐	☒ Antibodies
☐	☒ Eukaryotic cell lines
☒	☐ Palaeontology and archaeology
☐	☒ Animals and other organisms
☒	☐ Clinical data
☒	☐ Dual use research of concern
☒	☐ Plants

Methods

n/a	Involved in the study
☒	☐ ChIP-seq
☒	☐ Flow cytometry
☒	☐ MRI-based neuroimaging

Antibodies

Antibodies used

Western blot assay
The antibodies used for Western blotting included anti-Tau A-10 (sc-390476; Santa Cruz Biotechnology)
Goat Anti-mouse IgG(H+L)-HRP (1;1000; Southern Biotech; 1036-05; Lot#D4913-XC08D)
Elisa assay
Goat Anti-mouse IgG(H+L)-HRP (1;1000; Southern Biotech; 1036-05; Lot#D4913-XC08D)
Immunofluorescence assay
Alexa Fluor® 546-conjugated secondary antibody derived from goat anti-mouse IgG (Cat# A11003; Invitrogen)
Microglial uptake assay
Alexa FluorTM-555 conjugate (Invitrogen) and 4',6-diamidino-2-phenylindole (DAPI, Thermo Scientific) for visualization
Alexa FluorTM 594-conjugated donkey anti-rabbit secondary antibodies (1:500, Invitrogen, A-21207)
Immunohistochemical analysis
HRP conjugated anti-mouse secondary antibody (Cat# P0260, Dako)

Validation

All antibodies used in this study were validated by the suppliers as follows:
Western blot assay
The antibodies used for Western blotting included anti-Tau A-10 (sc-390476; Santa Cruz Biotechnology)
Manufacturer's website (https://www.scbt.com/p/tau-antibody-a-10)
For western blot and Elisa assay
Goat Anti-mouse IgG(H+L)-HRP (1;1000; Southern Biotech; 1036-05; Lot#D4913-XC08D)
Manufacturer's website (https://www.southernbiotech.com/goat-anti-mouse-igg-h-l-unlb-1036-01)
Immunoflurescence assay
Alexa Fluor® 546-conjugated secondary antibody derived from goat anti-mouse IgG (Cat# A11003; Invitrogen)
Manufacturer's website (https://www.thermofisher.com/antibody/product/Goat-anti-Mouse-IgG-H-L-Highly-Cross-Adsorbed-Secondary-Antibody-Polyclonal/A-11030)
Microglial uptake assay
Alexa FluorTM-555 conjugate (Invitrogen) and 4',6-diamidino-2-phenylindole (DAPI, Thermo Scientific) for visualization
Manufacturer's website (https://www.thermofisher.com/kr/ko/home/life-science/cell-analysis/fluorophores/alexa-fluor-555.html)
Alexa FluorTM 594-conjugated donkey anti-rabbit secondary antibodies (1:500, Invitrogen, A-21207)
Manufacturer's website (https://www.thermofisher.com/antibody/product/Donkey-anti-Rabbit-IgG-H-L-Highly-Cross-Adsorbed-Secondary-Antibody-Polyclonal/A-21207)
Immunohistochemical analysis
Manufacturer's website (https://www.citeab.com/antibodies/3288344-p0260-rabbit-anti-mouse-immunoglobulins-hrp-solid-p)

Eukaryotic cell lines

Policy information about cell lines and Sex and Gender in Research

Cell line source(s)	BV2 cells were generously provided by Dr. Changjong Moon of Chonnam National University (Gwangju, Korea)
Authentication	These cell lines were not Authenticated by us.
Mycoplasma contamination	We confirmed that cell line used was negative for mycoplasma contamination.
Commonly misidentified lines (See ICLAC register)	No commonly misidentified cell lines were used.

Animals and other research organisms

Policy information about studies involving animals; ARRIVE guidelines recommended for reporting animal research, and Sex and Gender in Research

Laboratory animals	Specific pathogen-free (SPF) female BALB/c mice were purchased from Orient Bio, Inc. (Seongnam, Korea).<br>BALB/c TLR5KO mice were previously characterized and described.<br>P301S-transgenic mice [B6; C3-Tg (Prnp-MAPT*P301S) PS19Vle/J]were obtained from the Jackson Laboratory.
Wild animals	This study did not involve wild animals.
Reporting on sex	Specific pathogen-free (SPF) female BALB/c mice(Female) were purchased from Orient Bio, Inc. (Seongnam, Korea).<br>BALB/c TLR5KO (Male) mice were previously characterized and described.<br>P301S-transgenic mice (Male) [B6; C3-Tg (Prnp-MAPT*P301S) PS19Vle/J]were obtained from the Jackson Laboratory.
Field-collected samples	This study did not involve animals collected from feild.
Ethics oversight	All animal experimental procedures were performed with the approval of the Chonnam National University Institutional Animal Care and Use Committee under protocol CNU IACUC 584 H-2022-45. The maintenance of the animal research facility and experimental procedures strictly adhered to the guidelines of the Animal Welfare Act enacted by the Korean Ministry of Agriculture, Food and Rural Affairs.

Note that full information on the approval of the study protocol must also be provided in the manuscript.

Plants

Seed stocks	n/a
Novel plant genotypes	n/a
Authentication	n/a
